# Evaluating cetacean body condition; a review of traditional approaches and new developments

**DOI:** 10.1002/ece3.6301

**Published:** 2020-05-13

**Authors:** Juliana Castrillon, Susan Bengtson Nash

**Affiliations:** ^1^ Southern Ocean Persistent Organic Pollutants Program Environmental Futures Research Institute (EFRI) Griffith University Nathan Qld. Australia

**Keywords:** Cetaceans, blubber measures, body condition, energetic health, individual fitness, Population monitoring

## Abstract

The ability to accurately gauge the body condition of free‐swimming cetaceans is invaluable in population and conservation biology, due to the direct implications that this measure has on individual fitness, survival, and reproductive success. Furthermore, monitoring temporal change in body condition offers insight into foraging success over time, and therefore the health of the supporting ecosystem, as well as a species’ resilience. These parameters are particularly relevant in the context of widespread and accelerated, climate‐induced habitat change. There are, however, significant logistical challenges involved with research and monitoring of large cetaceans, which often preclude direct measure of body condition of live individuals. Consequently, a wide variety of indirect approaches, or proxies, for estimating energetic stores have been proposed over past decades. To date, no single, standardized, approach has been shown to serve as a robust estimation of body condition across species, age categories, and in both live and dead individuals. Nonetheless, it is clear that streamlining and advancing body condition measures would carry significant benefits for diverse areas of cetacean research and management. Here, we review traditional approaches and new applications for the evaluation of cetacean energetic reserves. Specific attention is given to the criteria of measure performance (sensitivity and accuracy), level of invasiveness, cost and effort required for implementation, as well as versatility e.g. applicability across different species, age groups, as well as living versus deceased animals. Measures have been benchmarked against these criteria in an effort to identify key candidates for further development, and key research priorities in the field.

## Graphical abstract

Body condition in cetaceans has a direct implication on individual fitness, survival, and reproductive success, offering insight into their foraging success over time, and therefore the health of the supporting ecosystem and species resilience. However, to date, no single, standardized approach has been shown to be a robust estimation of body condition across species or age categories in both live and dead individuals. We review the traditional approaches and new developments for measuring energetic health in cetaceans, focusing in how versatile these approaches are across species, age, and life categories. In addition, this review will identify the capacity for high throughput analysis, which is necessary for application in routine monitoring.

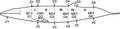

## 1 INTRODUCTION

The Body Condition (BC) of an animal is defined as the “relative size of its energy reserves” (Gosler, 1996; Krebs & Singleton, 1993; Schulte‐Hostedde, Millar, & Hickling, 2001; Schulte‐Hostedde, Zinner, Millar, & Hickling, 2005). BC is an important measurement of individual fitness, reflecting the balance between energy intake and total energetic investment (Green, 2001; Peig & Green, 2010; Schamber, Esler, & Flint, 2009).

Major energetic investments by mammals include courtship and mating, reproduction, lactation, and parental care, as well as migration or hibernation in certain species. Suboptimal BC in cetaceans has been shown to impact an individual's ability to fulfill these roles. Clear links have been observed between cetacean maternal BC and maternal investment and reproductive output (IWC, 2001; Lockyer & Waters, 1986; Williams et al. 2013). For example, low food availability, and consequently poor BC in killer whales (*Orcinus orca*), fin whales (*Balaenoptera physalus*), and southern right whales (*Eubalaena australis*) have been correlated with reduced calf numbers (Lockyer, 2007b; Ward, Holmes, & Balcomb, 2009; Williams et al. 2013; Seyboth et al., 2016). Further, negative repercussions of suboptimal maternal BC have been shown to be carried forward to the offspring. For instance, suboptimal maternal BC was observed to be associated with reduced fetal growth in fin and minke whales (*Balaenoptera acutorostrata*) (Lockyer, 2007a). Similarly, slower calf growth rate was observed in southern right whales and humpback whales (*Megaptera novaeangliae*) in response to lower maternal BC (Christiansen, Dujon, Sprogis, Arnould, & Bejder, 2016; Christiansen, Víkingsson, Rasmussen, & Lusseau, 2014; Christiansen et al., 2018). A lower calf birth and growth rate carries significant consequences for the viability and survival of offspring. In adults, individual BC not only affects female reproductive success, but also that of males. Higher energy reserves provide an advantage when defending or fighting for access to receptive females (Forsyth, Duncan, Tustin, & Gaillard, 2005; Lane, Boutin, Speakman, & Humphries, 2010; Toïgo, Gaillard, Laere, Hewison, & Morellet, 2006). Finally, in modeling studies, it is suggested that individual BC is an important predictor of behavior. Specifically, individuals with low energy reserves may be forced to increase their foraging effort, which could lead them to take greater risks, through, for example, greater predator exposure (Frid & Dill, 2002; Miller & Hall, 2012).

The direct repercussions of BC on individual and offspring fitness elevate the importance of effective BC evaluation for many areas of cetacean research and monitoring (Ryser‐Degiorgis, 2013). Additionally, as population BC is reflective of a population's foraging success, it can be indicative of ecosystem productivity, and over time, change (Braithwaite, Meeuwig, Letessier, Jenner, & Brierley, 2015; Harwood et al., 2015). As such, the role of BC as a sentinel parameter in ecosystem monitoring is receiving increased attention (Bengtson Nash et al., 2017; Tartu et al., 2017).

Evaluating the BC of marine mammals, particularly free‐swimming cetaceans, however, presents a unique suite of logistical challenges (Ball et al., 2017; Iverson, Sparling, Williams, & Shelley, 2010). Cetaceans spend the majority of their time under the water surface, and therefore obscured from sight, and with limited accessibility. While small cetaceans may be restrained temporarily, either in the wild or in captivity, the effort requires complex logistics in light of the highly mobile nature of these species. These challenges are magnified when targeting notoriously shy species, those occupying remote habitats, or critically endangered species. The high level of stress generated to the animal through capture warrants significant ethical considerations which, in the case of the latter, may preclude research permit allocation. As for larger cetaceans, their size and associated dangers of close interaction, preclude even temporary capture or restraint (Hunt et al., 2013).

Despite these challenges, the informative and predictive power of the BC parameter, have led to the development of a great number of techniques for quantitative estimation of BC in cetaceans. In the context of routine environmental monitoring, an ideal BC measure should be (1) sufficiently sensitive to ascertain differences in BC between individuals of the same species, as well as differences within individuals over time. The measure should further be, (2) Nonlethal, (3) inexpensive, both in terms of time and direct monetary costs, facilitating greater sample numbers. Finally, (4) the versatility of the measure in terms of its application to different categories, such as dead versus live individuals, carries consequences for the value and longevity of research and monitoring outputs derived with the BC measure. The following is an overview of published approaches, critically reviewed according to these four criteria.

## 2 BLUBBER MEASURES

Lipids, proteins, and carbohydrates are different forms in which mammals store energy. Lipids, with a higher energy density, are used as long‐term storage, while carbohydrates cover short term energy needs (Castellini & Rea, 1992; Hall et al., 2012; Robbins, 2012). Lipids represent the largest energy store. In cetaceans, lipid is primarily stored in the blubber tissue, a critical component of mammalian adaptation to the aquatic environment (Iverson & Koopman, 2018; Koopman, Pabst, McLellan, Dillaman, & Read, 2002). Aside from lipid storage (Ackman, Hingley, Eaton, Sipos, & Mitchell, 1975b; Iverson, 2002; Lockyer, 1987b; Parry, 1949), blubber also serves a multitude of physiological functions related to body hydrodynamics, water balance, buoyancy, and thermal insulation (Fish, 2000; Iverson & Koopman, 2018; Koopman et al., 2002; Ryg, Smith, & Øritsland, 1988). While some visceral lipid storage does occur (Víkingsson, 1995), the vast majority of lipids are stored in the blubber (Ackman, Hingley, Eaton, Sipos, et al., 1975; Iverson, 2002; Lockyer, 1987b; Parry, 1949). As such, the relative contribution of blubber to body mass is considered a reliable indicator of BC (Aguilar, Borrell, & Gómez‐Campos, 2007), and many methods have used this relationship for evaluating BC in cetaceans. Blubber‐metric methodologies are based on the inferred relationship between blubber volume, or blubber lipid content, and overall BC (Hanks, 1981; Schulte‐Hostedde et al., 2005). Key categories and approaches are outlined below.

### 2.1 Blubber mass

The most direct measure of blubber energy reserves is quantification of blubber mass, which is obtained through weighing of flensed blubber. This technique originates from commercial whaling, where, among other data, blubber mass was routinely obtained. The method has also been applied to incidentally caught and stranded animals and has provided useful baseline physiological information. For example, a study on harbour porpoises (*Phocoena phocoena*) killed incidentally during commercial fishing operations showed that the relationship between blubber mass and body size correlates with variation in BC among reproductive classes (Read, 1990), highlighting that calves and immature individuals were thinner than mature females. A similar study on incidentally caught franciscanas (*Pontoporia blainvillei*) found that blubber mass measurements strongly correlated with age class (Caon, Fialho, & Danilewicz, 2007). Today, a modification of the direct blubber mass approach is still used in whaling operations such as the Japanese Whale Research Program under Special Permit in the Antarctic (JARPA). This program uses “Fat weight” (blubber weight + visceral fat) of harvested Antarctic minke whales (*Balaenoptera bonaerensis*) as a BC indicator. Published reports from this program have evidenced an apparent decline in BC of harvested animals over two decades, with authors attributing the trend to reduced krill availability (Konishi et al., 2008).

Although blubber mass is the most direct quantification available for BC, it does not account for lipid content as a key measure of blubber quality. Total mass may reflect connective tissue and water, which have no bearing on BC. Additionally, blubber mass can only be applied to dead animals. While its application on stranded and incidentally killed animals may be valuable, harvesting animals for this purpose is usually in ethical conflict with research and monitoring agendas (Bateson, 1986; McMahon, Harcourt, Bateson, & Hindell, 2012; Waugh & Monamy, 2016). Furthermore, the technique is not without major logistical limitations. Dealing with a cetacean carcass is not an easy task, and as the animal size increases, specialized platforms and equipment become necessary (Lockyer, 1976).

### 2.2 Blubber thickness

A more accessible alternative to blubber mass is blubber thickness, which has also been used as a reflection of BC (Lockyer, 1987b; Víkingsson, 1995). Different methodologies have been developed based on this approach, and depending on the techniques applied, may provide a direct or indirect measure. Multiple site measures are advocated for better representation, as it is well known that blubber thickness is not homogenous across the body surface (Lockyer, McConnell, & Waters, 1984). As it not always is possible to take the measurement in different places of the same animal, particularly when working with free‐swimming individuals, it has been advocated that the measurement site be standardized to a site where the blubber is most variable (Lockyer, McConnell, & Waters, 1985b). In baleen whales, the dorso‐ventral region, posterior to the dorsal fin, represents the region of most variable blubber thickness and thus a good location for obtaining a measure correlative with total blubber mass (Aguilar et al., 2007; Konishi, 2006; Lockyer, 1987b). In smaller odontocetes, data suggest that this area corresponds to the anterior ventral region (Koopman, 2007; Zeng, Ji, Hao, & Wang, 2015).

#### 2.2.1 Direct measurement

Direct measurement of blubber thickness is carried out by cutting through the skin and blubber down to the muscle and measuring the full depth of the blubber. In its traditional application, it can only be applied to stranded or  harvested animals, or on small cetaceans that are temporarily restrained for surgical biopsy (Montie et al., 2008). Notably, direct measurement is more reliable on fresh carcasses due to normal postmortem decomposition changes (Zeng et al., 2015), which holds true for all approaches. Surgical biopsy is highly invasive when applied to live animals, not only because of the degree of stress inflicted  when restraining the animal but also due to the surgical wound caused by the procedure, which  have been observed to take a longer time to heal than wounds inflicted by remote biopsy (Weller et al. 1997).

Aside from ethical and logistical considerations, the accuracy of blubber thickness measurements may be impacted by the inherent loss of tension in the collagen matrix of the blubber tissue that occurs upon incision (Aguilar et al., 2007). Blubber tissue is a highly organized biocomposite, comprised of adipocytes in a three‐dimensional matrix of collagen and elastin fibers that maintain tissue tension (Toedt, 2001). Once the tissue is cut, it expands leading to a small but measurable increase in thickness (Aguilar et al., 2007). The degree of this increase may, in turn, be impacted by the tissue adipocyte/collagen proportion. Without factoring in this incremental change, and how it varies as a function of animal age and sampling season, it becomes difficult to investigate BC via this approach with any great confidence beyond its use as a relative measure between individuals.

#### 2.2.2 Indirect measurements

##### Ultrasound measurement

Ultrasound technology has provided an alternative way to measure blubber thickness, which has been shown to correlate with direct measurements (Cartee, Gray, John, & Ridgway, 1995; Zeng et al., 2015). It relies on the concept of sound traveling at different speeds through tissues of different density (Curran & Asher, 1974). This method has been more widely used in pinnipeds (Gales & Burton, 1987; Noren et al., 2015), for its ease of application when the animals are on land and can be immobilized. In cetaceans, it has been used on both stranded and free‐swimming animals. In stranded animals, it has the advantage of reducing the loss of lipids and tissue tension that occurs during necropsy. Additionally, it provides fast and valuable information on the distribution and structure of fat, enabling, for example, the identification of the different blubber layers present according to species type (Zeng et al., 2015). Also, for improved accuracy, as advocated with direct measures, measurements can easily be taken from different parts of the body.

In captive or free‐swimming cetaceans that can be temporarily restrained, the technique is similarly reliable. A mean measurement bias of 0.20 cm between ultrasound and direct measurement by ruler was reported in beluga whales (*Delphinapterus leucas*) (Cornick et al., 2016). The method has been successfully applied to captive harbour porpoises (Kastelein et al. 1995), bottlenose dolphins (*Tursiops truncatus*) (Cartee et al., 1995) and trialed on a single juvenile of a gray whale (*Eschrichtius robustus*) (Curran & Asher, 1974). The ideal scenario of restraining the animal for accurate measurements, however, limits its application to smaller cetaceans. Transfer of the technique to larger, free‐swimming cetaceans has, however, been attempted. For example, boat‐based ultrasound measurements were performed on free‐swimming right whales (Miller et al., 2011; Moore et al., 2001). Investigators noted both the need for further standardization of the protocol regarding sampling position on the body of the animal, as well as the difficulties associated with the operation of ultrasound equipment under boat‐based conditions (Moore et al., 2001). Advancements in the field of ultrasonography since this publication, including reduction in the size of ultrasound equipment, are helping to overcome the latter.

The above‐outlined blubber measures share common strengths and limitations. While the relevance of blubber in the study of cetacean BC is clear, the literature concerning the relationship between blubber thickness with BC is often conflicting and may vary between species, and within individuals over time (Aguilar et al., 2007; Caon et al., 2007; Dunkin, McLellan, Blum, & Pabst, 2005; Evans, Hindell, & Thiele, 2003; Gómez‐Campos, Borrell, & Aguilar, 2011; Kershaw, Brownlow, Ramp, Miller, & Hall, 2019; Read, 1990). A nonlinear relationship between BC and blubber thickness is driven by a number of factors. For example, lipid deposition and mobilization processes to and from lipid storage sites are highly dynamic and dependent upon the energetic state of the individual (Cropp, Bengtson Nash, & Hawker, 2014). If we consider that there is a succession to the dynamic, with visceral fat suggested as being more mobile than blubber stores; the “first in, first out” of energy stores (Lockyer et al., 1985a; Niæss, Haug, & Nilssen, 1998); then it is given that the overall BC blubber thickness relationship is only linear in a narrow range. That is, that significant lipid energy depletion can occur in a very good BC individual, without noticeable change in blubber thickness.

The ancillary role of blubber in thermoregulation further  confounds the BC‐blubber thickness relationship. The surface area to volume ratio and the thermal environment, determine the heat loss of an individual (McLellan et al., 2002; Worthy & Edwards, 1990). To cope with varying thermal regimes, cetaceans may adapt the insulative properties of the blubber tissue; both blubber quantity (thickness) and blubber quality (lipid content) (Dunkin et al., 2005; Kvadsheim, Folkow, & Blix, 1996; Worthy & Edwards, 1990). In light of such confounding factors, alternate or supporting BC measures to blubber thickness are advocated in lieu of species‐specific investigation of the relationships.

### 2.3 Blubber lipid content

A change in the overall energy stores of cetaceans is reflected not only in the thickness of the blubber tissue, but also the composition of the blubber tissue, particularly its lipid content (Aguilar & Borrell, 1990). As such, the lipid content of blubber has been proposed as a measure of BC in cetaceans (Aguilar & Borrell, 1990; Krahn et al., 2001).

Quantification of total blubber lipid content is feasible only for carcasses and has its origin in the whaling industry where “oil yields” were measured. Most recently,  historical  measures from humpback and sperm whales (*Physeter macrocephalus*) (Irvine, Thums, Hanson, McMahon, & Hindell, 2017) were used to , draw links between the interannual BC, as interpolated from annual oil yields, and krill densities in the corresponding Antarctic feeding grounds of the population (Braithwaite et al., 2015). Despite the valuable information gained from this data, comparisons and conclusions must be made with caution because of the inconsistency of sample type; some sets of data refer to the amount of oil extracted exclusively from the blubber, whilst others  report oil extracted from the entire carcass. The data also differ in the way in which the oil yield was recovered. For instance, some records were detailed enough to report the oil yield at an individual level; however, as the industry grew, oil yields were reported as e.g.  weekly tallies. Finally, falsification of whaling data is widely reported throughout history, and caution must be taken when interpolating quantitative measures from these forms of data (Clapham & Ivashchenko, 2018).

Total blubber lipid content measures can inherently only be obtained from dead animals, and this approach shares the afore‐mentioned logistical limitations associated with quantification of blubber mass. An adaptation of the technique whereby lipid percent by blubber mass is determined, has, however, been routinely used as an indicator of BC. As the approach requires only a small blubber sample, it can be applied to both dead and free‐swimming animals (Beck, Smith, & Hammill, 1993; Kershaw et al., 2019; Shier & Schemmel, 1975; Stirling, Thiemann, & Richardson, 2008). While necropsy of dead individuals provides greater access to blubber tissues of varying depth, body location, and samples size, samples from free‐swimming individuals are readily obtained through remote biopsy. The use of biopsy darts for the remote collection of tissue samples from free‐swimming cetaceans has gained popularity in the recent past due to the nonlethal and minimally invasive nature of the method (Noren & Mocklin, 2012). For most research and monitoring efforts, remote biopsy samples from presumably healthy animals are preferred over samples collected from stranded individuals as stranded individuals often represent the very young, old or diseased animals and are therefore  not representative of the overall population (Aguilar, Borrell, & Pastor, 1999; Krahn, Herman, Ylitalo, Sloan, & BURROwS D.G., Hobbs R.C., Mahoney B.A., Yanagida G.K., Calambokidis J. & Moore S., 2004a).

Blubber  lipid quantification involves extraction of lipids from a pre‐weighed blubber sample (Varanasi et al., 1994). As lipid content is also an important factor in other areas of cetacean research, such as evaluation of lipophilic contaminant burdens, its advantage is that it is already widely integrated into many monitoring programs. Nevertheless, as with blubber thickness, information derived via the lipid % measure with regard to BC is often conflicting. While Bengtson Nash, Waugh, and Schlabach (2013a) found a significant reduction in outer blubber lipid % between fed and fasted cohorts (*n* = 58) of adult, male, southern hemisphere humpback whales, paired morphometric UAV measures and outer blubber lipid % measures of 9 mixed gender adults and 16 mixed‐gender juveniles did not show a linear relationship (Christiansen et al.^2020^). Similarly, Evans et al. (2003) found no correlation between blubber thickness and blubber lipid content of stranded sperm whales (*n* = 108). Most recently, Kershaw et al. (2019) found no correlation between lipid content and blubber thickness of stranded humpback whales (*n* = 3), sowerby´s beaked whale (*Mesoplodon bidenis*) (*n* = 4), cuvier´s beaked whales (*Ziphius cavirostris*) (*n* = 2) nor northern bottlenose whale (*Hyperoodon ampullatus*) (*n* = 2).

The major weaknesses of the blubber lipid % measure relate to blubber stratification, specifically the outer blubber layer, as well the high level of variability introduced through sample acquisition and lipid analysis. Blubber is a complex tissue and is not homogenous throughout its depth in neither composition of function (Koopman, Iverson, & Gaskin, 1996b; Krahn, Herman, Ylitalo, Sloan, Burrows, et al., 2004). The outer blubber layer, in particular, serves a number of ancillary functions aside from lipid storage. These include thermoregulation, buoyancy, water balance, and locomotion (Koopman et al., 2002; Montie et al., 2008; Ryg et al., 1988; Strandberg et al., 2008). It is given, therefore, that there exists a limit to the amount of lipid that may be lost from this layer without jeopardizing these ancillary functions, and therefore individual survival (Evans et al., 2003; Gómez‐Campos et al., 2011; Waugh, Nichols, Noad, & Bengtson Nash, 2012; Noren et al., 2015; Ball et al., 2017; Castrillon et al. 2017; Bengtson Nash, 2018b). All measures targeting the outer blubber layer thus share what we have termed the *Outer Blubber Layer Threshold* limitation that may be summarized as a resistance to loss of maintainance lipids from this layer across normal BC ranges.

While in some cetaceans, such as bottlenose and common dolphins (*Delphinus delphis*), harbour porpoise, sei whales (*Balaenoptera borealis*), and fin whales, blubber is stratified in well‐defined layers (Ackman, Hingley, Eaton, Logan, & Odense, 1975a; Aguilar & Borrell, 1990; Koopman et al., 1996a, 2002; Lockyer et al., 1984; Montie et al., 2008; Samuel & Worthy, 2004), in other species including humpback and bowhead whales (*Balaena mysticetus*), the transition between outer to inner blubber is gradual (Ackman, Hingley, Eaton, Logan, et al., 1975; Ball et al., 2015; Elfes, 2008; Waugh, Nichols, Schlabach, Noad, & Bengtson Nash, 2014). The inner layer is thought to be the most metabolically active, in terms of lipogenesis and lipolysis (Olsen & Grahl‐Nielsen, 2003) with a fatty acid composition that is strongly affected by most recent lipid mobilization/deposition processes. Adipocytes in this layer are high in number but often small in size. The middle layer is used for lipid storage, with often larger but fewer adipocytes (Koopman et al., 2002; Montie et al., 2008; Ryg et al., 1988; Strandberg et al., 2008).

Error and variability of blubber lipid% measures introduced through sampling relate to uncertainty regarding the blubber layer captured, as well as lipid loss through excision. Uncertainty regarding blubber layer is easily controlled during necropsy where visual and biochemical assessment of the full blubber layer is possible. On the other hand, remote biopsy leaves substantial room for uncertainty. Not only does the biopsy penetration depth depend on factors such as the dart head used, the pressure and angle with which the dart penetrates the tissue, but little is ever known regarding what proportion of the total blubber tissue, the sample portion represents.

As soon as blubber is cut, lipid will leak from the tissue. The error that this lipid loss introduces to lipid % calculations will depend on both the size of the tissue sample used for lipid analysis,  with smaller sample masses yielding a higher proportional error, as well as the efficiency of tissue processing upon collection, for example the duration of  submersion in seawater, and until storage.  Studies have shown that blubber lipid content from remotely biopsied tissues was not representative of directly harvested blubber tissue (Krahn, Herman, Ylitalo, Sloan, Burrows, Hobbs, Mahoney, Yanagida, & Calambokidis, & Moore, 2004; McKinney et al., 2014; Ryan, McHugh, O’Connor, & Berrow, 2013). A difference of up to 44% in lipid content was found between comparable samples taken by biopsy and excised using a scalpel in fin whale blubber (Ryan et al., 2013).

Finally, the lipid % measure has been derived  from a wide variety of, frequently unspecified, analytical protocols used for lipid extraction. Typically, some variation of a chloroform–methanol solvent extraction is used (Bligh & Dyer, 1959; Budge, Iverson, & Koopman, 2006; Folch, Lees, & Sloane‐Stanley, 1957; Smedes, 1999). Occasionally, however, stronger solvent mixes, such as hexane, acetone, and dichloromethane are used for extraction (Casa et al. 2019). All the lipid content measures assume first that the lipid % of a blubber sample is representative of the body region from where it was obtained, and second, that the extraction method efficiently strips all, and only, lipids from the blubber (Ryan et al., 2013). Cruder extraction methods will strip additional organic material from the tissue, representing a  methodological error and introducing inter‐study variability.

### 2.4 Blubber trunk lipid mass (BTLM)

Blubber trunk lipid mass (BTLM), a hybrid measure of blubber mass and lipid content, has been proposed as an index for blubber mass (Gómez‐Campos et al., 2011). The trunk of many cetaceans is a region of highly dynamic lipid deposition and mobilization (Lockyer, 1987a), and hence a body region more likely to reflect a linear change in response to BC. BTLM is derived by considering the total amount of lipid stored in the trunk blubber mass, and it is calculated by the following formula:

BTLM(Kg)=%lipidsinblubber×blubber weight

A study on striped dolphins (*Stenella coeruleoalba*) showed that the BTLM measurements exceeded the accuracy for blubber lipid content assessments (Gómez‐Campos et al., 2011). The major limitation of this measure is the fact that it is only applicable to dead animals and only individuals and species  of a manageable size.

### 2.5 Adipocyte metrics

**Figure 1:**
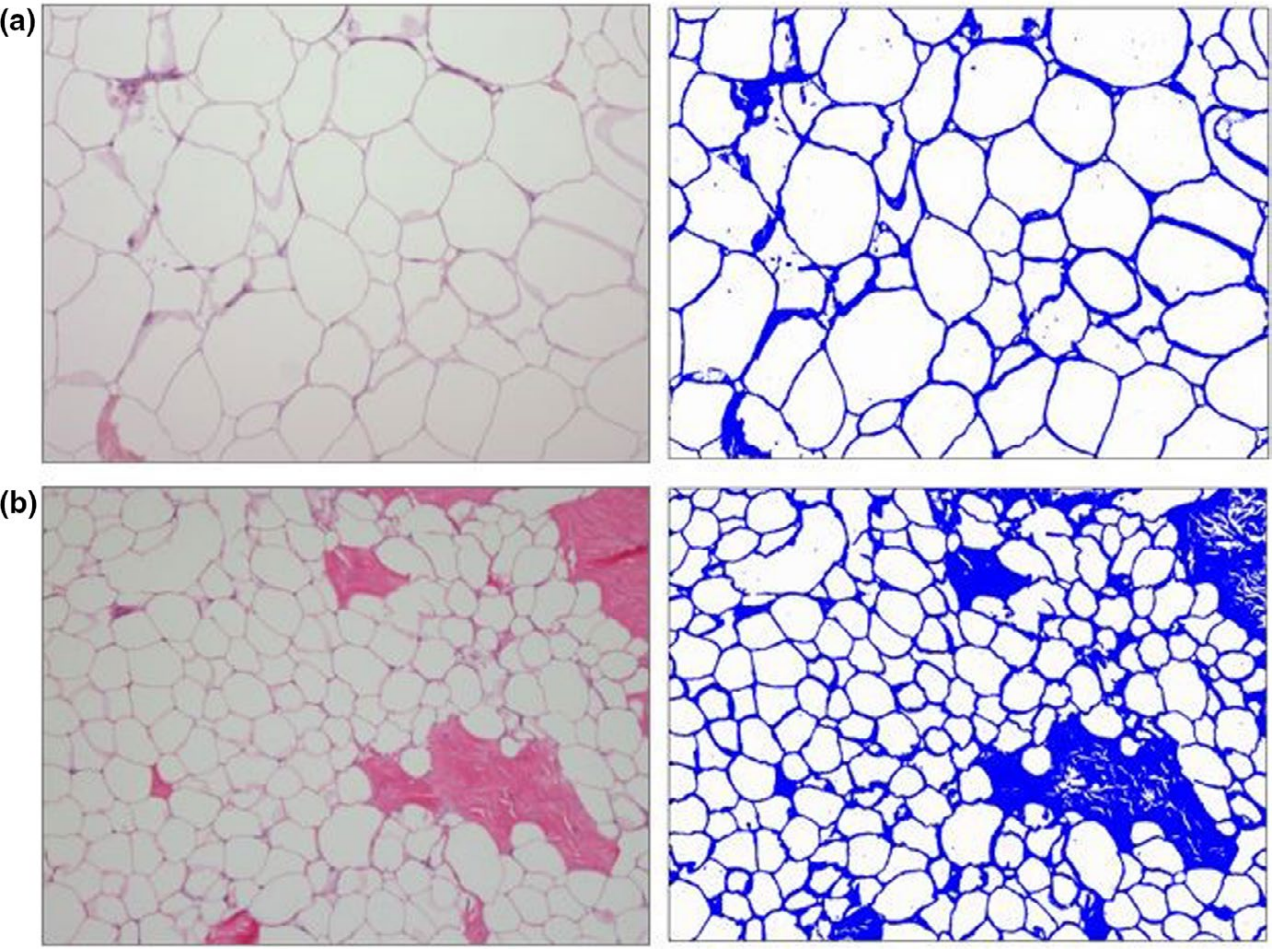
Histological (hematoxylin & eosin stain) and AI analysis images of blubber from two individual humpback whales (a and b) with different BC. (a) AI = 0.16. (b) AI = 0.45

The measurement of relative adipocyte volume has been proposed as a proxy of BC. While adipocyte histology has previously been used to make BC evaluations in, for example, harbour porpoises (Koopman et al., 2002), an effort has recently been made to develop a standardized approach (Castrillon et al. 2017). The concept behind using adipocyte area or volume as a proxy for BC lies in the fact that very early on in mammalian development, the number of adipocyte cells is set so that a change in BC will be reflected in an increase or decrease in adipocyte volume, as opposed to adipocyte number (Faust, Johnson, Stern, & Hirsch, 1978). The original approach measured the adipocyte area from histologically prepared images of blubber tissue (Castrillon et al. 2017). The tissue is stained, to differentiate the adipocyte cells from the collagen matrix. Adipocyte area is calculated as the average adipocyte cross‐sectional area from measurements of at least 100 individual adipocytes. Given the laborious nature of this method, rendering it impractical for high‐throughput, routine analysis, an index measure derived through automated image analysis, was developed. The Adipocyte Index (AI) is defined as the ratio of inter‐vacuolar area to adipocyte area within a defined image area (Figure 1). To make the index more intuitive, that is, a larger value for better BC, the AI^−1^ was later introduced (Druskat, Ghosh, Castrillon, & Bengtson Nash, 2019).

Application of adipocyte metrics in a study on humpback whales found that the measures were more sensitive in differentiating between two cohorts of whales at different stages of fasting than blubber lipid content. Specifically, AI and adipocyte area differentiated the two cohorts, whereas blubber lipid content failed to do so.

This approach is suitable for application in live or dead animals, although the freshness of tissue is of some importance. The method is subject to the Outer Blubber Threshold limitations discussed above, which also carry some consequence for age cohorts. It is for example not advisable to perform analysis on calves or juveniles where the blubber tissue is not yet fully developed.

## 3 BODY COMPOSITION

Several approaches for BC quantification have used body composition analysis. Body composition analyseis approaches focus on the categorization of body mass into major body components, identified as water, fat, protein, carbohydrates, and inorganic constituents, based on each component's physical properties (Boyd, Bowen, & Iverson, 2010; Speakman, 2001). The total body composition analysis measures the total mass of each of these components in the body. The Total Body Fat (TBF) measurement of this approach is taken as representative of BC. Several body composition estimation approaches have been applied to cetaceans.

### 3.1 Carcass analysis

The gold standard for body composition analysis is whole carcass analysis, and therefore, the transfer of the technique to tissues biopsied from live individuals is not considered to be as accurate (Wells & Fewtrell, 2006). Carcass analysis can be carried out by bomb calorimetry or fat mass estimation and is performed on the whole carcass or of subsamples of the homogenized carcass (Iverson et al., 2010). The bomb calorimetry approach measures the calorimetric content of the homogenized carcass. This measure can be used to determine the fat and protein content, which in turn can be transformed to a body energy equivalent using fat and protein density standard values, 39.5 MJ/kg and 23.5 MJ/kg, respectively (Boyd et al., 2010; Schmidt‐Nielsen, Bolis, & Taylor, 1980). Fat mass estimation is determined by taking the difference between the dry mass of the sample before and after lipid extraction (Speakman, 2001). While measurements can also be made on a tissue‐specific basis, as is done with blubber lipid content, results do not correlate with whole‐body composition but rather with tissue‐specific estimations. The need to grind the whole body of the animal limits these applications not just to dead animals, but also to small species or juveniles (Boyd et al., 2010). Despite the clear challenges of this approach, whole body estimates have been obtained for fin, sei, and sperm whales as well as harbour porpoises, providing important baseline information regarding lipid content, protein, and ash content (Lockyer, 1991; Lockyer et al., 1984, 1985b; McLellan et al., 2002).

### 3.2 Isotope dilution

Nonlethal techniques have also been developed to determine body composition. Bioelectrical Impedance Analysis (BIA) and isotope dilution are two of these techniques. These approaches do not measure body composition directly, but rather infer it from measurements of body properties (Wells & Fewtrell, 2006). Both techniques use the measure of Total Body Water (TBW) to predict the TBF in an individual. As water is not evenly distributed in the body tissues, with fat tissue containing substantially less water than lean tissue (Speakman, 2001). The fatter an organism becomes, the lower the water content as a percentage of its total body mass (Speakman, 2001). Both techniques have been carried out on pinnipeds for BC evaluation (Arnould, 1995; Bowen & Iverson, 1998; Reilly & Fedak, 1990), but only isotope dilution has been applied to cetaceans, focusing on research questions related to specific physiological functions such as osmosis, water consumption and flux, rather than BC (Hui, 1981; Telfer, Cornell, & Prescott, 1970).

For this reason, isotope dilution is briefly presented as part of this review as a potential approach for evaluating BC of live, captive individuals. For a more detailed explanation of this technique, refer to Speakman (2001), or for a more specific application on seals, to Schwarz et al. (2015). Briefly, isotope dilution requires the injection of a known dose of isotope labeled water (D₂O, H₂^1^⁸O or ^3^H₂O) into live animals (Smith, Engel, Diskin, Španěl, & Davies, 2002). After allowing a period of equilibration of the labeled water within an animal, blood, urine, or saliva samples are collected at specific intervals to develop a dilution curve, quantified by stable‐isotope mass spectrometry (Castellini & Mellish, 2015). It has been established that the water content of lean tissue is approximately 73% (Pace & Rathbun, 1945) and based on this value, it is possible to calculate TBW. Initially, it was thought that these values were relatively stable; but now is known that individual and population variation can be large. For instance, young animals have lean tissues with higher water percentage (Sawicka‐Kapusta, 1974). The uncertainty regarding the absolute value of water content in lean tissue is undoubtedly the biggest problem when estimating TBF, using the isotope dilution technique (Speakman, 2001). For this reason, body‐size, age‐, and sex‐specific equations still need to be developed prior to method implementation. The approach shares the limitations of the need to restrain the animal for a period of time, for weighing and sample collection, limiting its application to small species and captive animals. The high cost and health risks associated with the use of radioisotopes (^3^H) require special permits, equipment, and waste disposal, further increasing the logistical challenges associated with this technique.

### 3.3 Glide method

A more recent indirect approach for predicting BC is that of using glide to determine body density. Glides are the periods of a dive where the individual is not actively fluking. During these nonactive swimming phases, the forces of drag and lift are acting on the body in a way that is dependent on overall body density/buoyancy. As lipids are less dense than most other tissues (Biuw, McConnell, Bradshaw, Burton, & Fedak, 2003), TBF is directly related to body density. Body density determines the rate of speed changes during glides (Miller et al., 2016), with denser animals found to glide at a slower speed (Boyd et al., 2010).

A data logger with 3‐axis acceleration and speed sensors is attached to the animal. A glide model uses the data from this device to recreate the dive profile, allowing calculation of deceleration/acceleration from glide speed data. The acceleration during a glide is the difference between the drag forces and the net buoyancy along the individual swimming trajectory (Miller, Johnson, Tyack, & Terray, 2004; Zhang et al., 2019). To validate this technique directly with BC, estimates of relative lipid content of individual seals obtained by glide pattern analysis were compared with those obtained by hydrogen isotope dilution, with a variation in the results of about ± 2% (Biuw et al., 2003). Validation of the technique, in a preliminary study in cetaceans, was not, however, made with a quantitative measure of BC, but rather modeled estimates (Miller et al., 2016). In this study, it was reported that the obtained results fit the model with good precision in the deep‐diving cetacean species, northern bottlenose whales (*Hyperoodon ampullatus*) (Miller et al., 2016). The model, however, needed adjustments to account for diving air volume in shallower diving cetacean species, such as humpback whales, as the effect of ambient air pressure on animal density is reduced by compression at depth (Biuw et al., 2003; Miller et al., 2016). In cetaceans, the glide model approach has also been applied to sperm whales, albeit to describe the swimming behavior rather than evaluate BC (Miller et al., 2004). To apply the glide model to humpback whales, it was modified to consider the effect of the air volume in the net buoyancy, and the potential effect of the drag induced by lift. Humpback whales tend to dive and glide at alternative, shallower pitch angles requiring the generation of lift in comparison to deep‐diving cetaceans that maintain steep pitch angles during glides (Narazaki et al., 2018). The study concluded that the glide method has the potential to estimate BC in shallow diving baleen whales despite results being more precise in deep‐diving toothed whales.

The glide method carries the logistical challenges of tagging but also the distinct advantage that the glide measure reflects total body fat. The approach has not yet been completely validated for its use as a proxy for BC. Application of the approach would further require species‐specific evaluation and optimization of the model used due to the variability in the diving behavior across species (Miller et al., 2016).

## 4 BODY MORPHOMETRY

Another approach used as a proxy for BC is morphological measurements to infer whole body mass. Morphometry is the numerical expression of animal morphological characteristics (Stower, Davies, & Jones, 1960). Complications associated with the direct weighing of carcasses have led to the development of a significant number of BC indices derived from morphological measurements which, downstream, also predict body mass (Boyd et al., 2010; Cattet, Atkinson, Polischuk, & Ramsay, 1997). All approaches assume the cetacean species has an ellipsoid shape and that a dependent relationship exists between BC and body mass (Jakob, Marshall, & Uetz, 1996; Peig & Green, 2010). The indices are developed according to empirical measurements, of which the most common are girth, and girth in relation to body length.

### 4.1 Body girth

Body girth measurement data have been used both in isolation, and for deriving BC indices (Gómez‐Campos et al., 2011; Lockyer & Waters, 1986). By convention, the measurement is taken from the front of the dorsal fin, where the animal's girth is at its maximum (Boyd et al., 2010). Contradictory results have been found regarding the consistency of the measurement as a reflection of the BC. In franciscanas (Caon et al., 2007), and minke whales (Konishi, 2006) body girth, blubber weight, and body weight were all found to be positively correlated, lending support for its value as a BC measure. Similarly, in fin (Lockyer, 1986) and bowhead whales (George, Druckenmiller, Laidre, Suydam, & Person, 2015), significant differences in body girth were found between reproductive groups. By contrast, body girth was poorly correlated with blubber mass in striped dolphins (Gómez‐Campos et al., 2011) and harbour porpoises (Read, 1990).

In addition to BC, the overall body size and thus the age of the animal is an aspect that also affects body girth; therefore, this needs to be accounted for when using this method and comparing individuals. Although this measure is taken routinely on captive, live captured and stranded animals, the need to handle the animals, limits its application to small species. Several factors can further influence the measure. For example, in stranded animals, the degree of bloating and decomposition may impact results (Boyd et al., 2010). In live animals, in addition to the difficulties associated with capturing and handling of a wild animal, the measurement may vary with pregnancy, and even with small animal movements, such as breathing (Lockyer *et al.* 2003).

### 4.2 Body girth—length

The relationship between body girth and body length has been applied in a variety of ways to predict body volume. This has also been used as a proxy of BC due to its close correlation with body mass. The girth to length ratio (Ichii, Shinohara, Fujise, Nishiwaki, & Matsuoka, 1998; Kershaw, Sherrill, Davison, Brownlow, & Hall, 2017), girth—length regression (Lockyer & Waters, 1986), and residuals from girth—length regression (Haug, Lindstrøm, & Nilssen, 2002) are among some of the calculations applied, either in isolation to estimate body volume, or as part of more complex BC indices. However, it is important to note that the relationship is species and age‐category specific, with anomalies found particularly among very young individuals that are still growing in length. Immature individuals allocate a significant amount of energy toward growth rather than building fat reserves (Peig & Green, 2010). A leaner juvenile, as measured by body girth and length, may, therefore, be in better energetic health than an adult with comparable, or even better, BC.

Extensive literature exists on the selection and calculation of various BC indices using body morphometry metrics. For recent reviews, see Peig and Green (2010), Labocha and Hayes (2012) and Labocha, Schutz, and Hayes (2014). As such, this review will not focus on reviewing the benefits and drawbacks of individual indices, but rather the collection of techniques for obtaining the empirical morphometry data used for BC index calculations. Some general considerations regarding the use of BC indices based on morphometric data are, however, warranted. It should be noted that, to date, there is no clear consensus on whether these indices are sufficiently accurate or sensitive, nor the range of circumstances under which they may be valid (Cook et al., 2001). Further, it is advocated that any BC index should include nonmorphological parameters that are known to influence blubber variation, such as sex, age, reproductive class/state, day in the feeding season, and stage of the annual reproductive cycle to improve the indices’ accuracy (Boyd et al., 2010; Christiansen et al., 2014).

### 4.3 Body volume models

A truncated cone model is the most commonly used methodology when calculating body volume, especially in pinnipeds. The method models the external morphology of marine mammals as a series of cylinders and conical frustrums. (Bell, Hindell, & Burton, 1997; Ryg et al., 1988; Luque & Aurioles‐Gamboa 2001). However, cetaceans have highly streamlined body shapes that are not likely to be well represented by a series of cones and cylinders. Therefore, 3D modeling may provide a more accurate representation of external cetacean morphology (Adamczak, Pabst, McLellan, & Thorne, 2019). Such modeling was performed in two species of pilot whales, short‐, and long‐finned pilot whales (*Globicephala macrorhynchus and G. melas*), as a preliminary study. A baseline model of the core body was created using morphometric measurements and digital photographs, with a 3D mesh around the body. See details of modeling construction in Adamczak et al. (2019).

Using morphological measurements from stranding data; specifically several girth measurements along the body, as well as body length, the base model was scaled and modified to represent the specific external morphology of each whale in the sample, accounting for morphological differences between individuals. Both, the truncated cone method and 3D model were performed with the same set of data. The 3D model better represented the external morphology of pilot whales, particularly in the tail stock region where the truncated cones method failed to account for its sharp elliptical cross‐sectional shape, yielding anomalously high superficial area and volume values.

The 3D model assessment was done visually, and no further validations with actual body volume have been performed. The model was applied only on mature females and males, with pregnant and lactating females, and immature individuals excluded. To date, the proxy has only been applied only to stranded animals.

**Figure 2:**
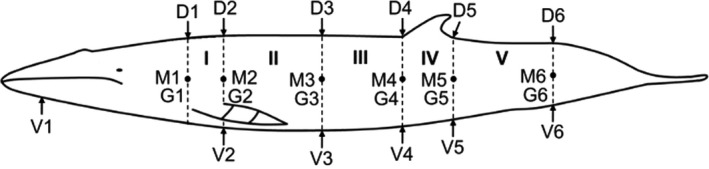
Measuring sites for blubber thickness and girth measurements. G1–G6 are the girth measurement positions. D1–D6, M1–M6, and V1–V6 are the dorsal, medial, and ventral sites where blubber thickness was measured. The different body sections used for the frustum volume estimations are marked with roman numerals I–V. Figure replicated from Christiansen et al. (2013)

Another example of body volume modeling is presented by Christiansen, Víkingsson, Rasmussen, and Lusseau (2013), with particular attention to blubber volume. The total blubber volume was estimated in minke whale harvested in Iceland using multiple direct measurements of blubber thickness, girth, and length (Christiansen et al., 2013) (Figure 2). Again, the body modeling was carried out as a series of frustrums, to take into consideration the variation in girths between measurements sites. The study found an increase in blubber volume during the feeding season, in mature and pregnant whales, but not in immature whales, likely due to preferential energy investment into growth in these animals.

Body morphometry models developed on stranded and harvested animals carry great potential to be transferred to free‐swimming individuals, using photogrammetry methods described in subsequent sections, where benefits and limitations of the approaches are also further discussed.

### 4.4 Photogrammetry

Due to the challenge of measuring different body parts in free‐swimming and in stranded individuals, the application of body morphometry measures using photographic images of the individual (photogrammetry) to measure different parts of the body has become a popular approach (Best & Rüther, 1992; Cubbage & Calambokidis, 1987; Durban, Fearnbach, Barrett‐Lennard, Perryman, & Leroi, 2015; Koski, Rugh, Punt, & Zeh, 2006; Whitehead & Payne, 1981). As the data obtained with photogrammetry is two‐dimensional, the approach uses width measurements taken along the body to calculate body shape (Burnett et al., 2018; Christiansen et al., 2014, 2018; Miller, Best, Perryman, Baumgartner, & Moore, 2012).

Most of the photogrammetry techniques, such as stereo‐photogrammetry (Bräger & Chong, 1999; Brager, Chong, Dawson, Slooten, & Wursig, 1999; Cubbage & Calambokidis, 1987), laser‐photogrammetry (Clarke, Aguayo, & Obla, 1972; Durban & Parsons, 2006; Jaquet, 2006; Webster, Dawson, & Slooten, 2010) and underwater‐videography (Nolan & Liddle, 2000), have been used to determine the body size of the animal, either directly or indirectly. Few have been applied for the specific purpose BC evaluation. The exception to this is aerial‐photogrammetry.

#### 4.4.1 Aerial‐photogrammetry

In the traditional application, individuals at the surface of the water are photographed from an aeroplane or helicopter at a known height. In general, a known altitude and lens focal length is used to scale the image (Miller et al., 2016). BC indices have been calculated in gray (Perryman & Lynn, 2002) and right whales (Miller & Hall, 2012), using body measurements from photographs. However, the high costs of manned aerial photographs are one of the major drawbacks of this approach.

**Figure 3:**
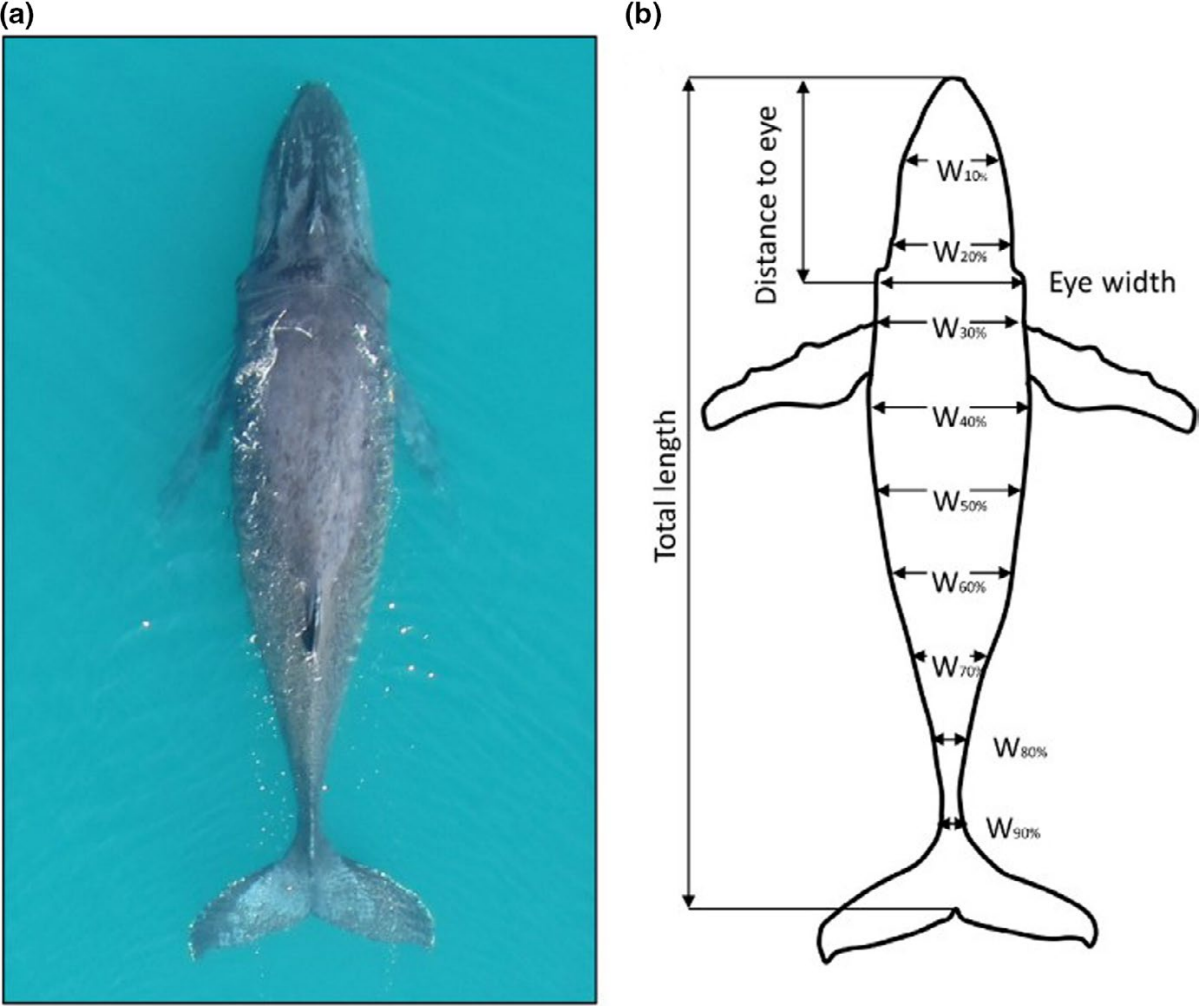
(a) An example of a desired aerial photograph of a humpback whale captured by an unmanned aerial vehicle. The whale is lying flat at the surface, dorsal side facing up, with a straight body axis and peduncle (nonarching). (b) Position of measurement sites of humpback whales proposed by Christiansen et al. (2016). For clarity, only width (W) measurement sites located at 10% increments along the body axis are shown. Image replicated from Christiansen et al. (2016)

In recent years, researches have taken advantage of the rapid developments of Unmanned Aerial Vehicle (UAV) technology. UAV‐derived photogrammetry images have been used to determine BC of humpback (Christiansen et al., 2016), right (Christiansen et al., 2018), gray and pygmy blue whales (*Balaenoptera musculus brevicauda*) (Burnett et al., 2018), by taking vertical aerial photographs of individuals swimming at the surface (Figure 3). Photographs are scaled by images which include both the target animal and the research vessel (Christiansen et al., 2016), or imaging an object of known length every flight (Burnett et al., 2018). The UAV‐based approach significantly reduces disturbances to animals, is a much safer approach for researchers and greatly reduces the costs of sampling. While aerial photography has traditionally been incredibly expensive, being only accessible to some researchers, the cost of UAVs is continually decreasing, making the approach more accessible on a broader scale and thus implemented in many areas of research. Another advantage of this approach is the simplified implementation of aerial photography. Previously, it was necessary to have a specialized team, including an aeroplane pilot, and experienced photographer with large specialised cameras and lenses. UAVs combine all the major equipment and the sampling can be carried out by a small team.

Considerations for the application of morphometric measures from aerial photographs include both those related to animal physiology, as well as technical considerations. For example, any BC measure that focuses on body volume measurements must consider the exceptions and anomalies introduced above, such as pregnancy and lung volume. Reported technical error sources relate to image distortion, picture quality, and measurement precision. Environmental factors, such as glare, waves, water spray, and turbidity, can distort the animal contour, reducing accuracy (Christiansen et al., 2016). Picture quality refers to the position of the animal in the photograph, with an image of the individual in a straight line in order to calculate its length accurately. Measurement precision error can be reduced by using independent researchers measuring the same set of photographs. The latter can add labor and cost to processing of images, however, the recent development of a *Whale Quantitative Analysis* program in R by Burnett et al. (2018) is expected to improve processing efficiency.

## 5 BIOCHEMICAL AND CHEMICAL BIOMARKERS OF BC

A biomarker is a naturally occurring molecule, gene, or characteristic by which a pathological or physiological process can be identified (Atkinson et al., 2001). The application of biomarkers is widely used in areas such as medicine, ecotoxicology, and ecology. While the identification and development of reliable biomarkers requires detailed knowledge of complex physiological and biochemical processes, their identification and validation can yield powerful tools for health and ecosystem monitoring, including BC.

### 5.1 Lipophilic contaminant concentration index (CI)

The use of lipophilic, Persistent Organic Pollutants (POPs) burdens has been proposed as suitable biomarkers of fluctuating BC in humpback whales (Bengtson Nash, Waugh, & Schlabach, 2013b; Bengtson Nash 2018a). POPs are synthetic compounds defined by their persistence, toxicity, propensity to bioaccumulate in organisms, and their capacity for long‐range environmental dispersal. Most known POPs are lipophilic, accumulating in lipid‐rich tissues of organisms, with their toxicokinetics being driven by lipid dynamics (Bengtson Nash et al., 2013b; Yordy et al., 2010). Humpback whales in the Southern Hemisphere undertake annual migrations involving voluntary fasting for four to nine months. Investigators previously found that across just four months of the migration journey, POP concentrations in the outer blubber layer increased by up to 500 times for some compounds (Bengtson Nash et al., 2013b), and utilized the chemical Concentration Index (CI) to demonstrate this effect. Lipid loss from the blubber alone could not explain this increase; hence, the CI demonstrated whole‐body lipid depletion and remobilization of associated POP burdens (Bengtson Nash, 2018b). As the lipid reserves are converted to energy, but the POP burdens are unable to be metabolized, the POPs merely redistribute to the body's remaining lipid depots, of which the outer blubber layer starts to represent an increasing proportion due to the Outer Blubber Threshold effect (Bengtson Nash et al., 2013b).

Although this approach has been used to measure change in population BC over time, the approach would be equally valid for individuals tracked over time, or similarly defined populations. The approach is particularly suitable for polar foraging species where contaminant sources are diffuse and not influenced by localized emissions. The approach could not, however, be used to compare two diverse populations with different diets where direct contaminant uptake differences may contribute significantly to observed differences in blubber POP burdens. Similarly, diet‐associated factors could not be excluded in populations with a high level of individual exchange with other populations. Finally, the cost of POP analysis is notoriously expensive so while information regarding BC may be valuable supplementary information to any long‐term POP monitoring program, a focus on POPs purely for BC investigation may be considered prohibitively expensive.

### 5.2 Omics

Advances in molecular sequencing, in addition to both chemical and biochemical detection techniques, offer the potential for accelerated identification of suitable biomarkers of BC. Specifically, transcriptomics, proteomics, and metabolomics applied in phocid species have flagged their potential for application in cetaceans. This technology is primarily aimed at the universal detection of mRNA (transcriptomics), proteins (proteomics), and metabolites (metabolomics) in a specific biological sample in a nonspecific and unbiased way (Horgan & Kenny, 2011). Transcriptome sequencing offers an insight into which genes were active (being transcribed) at the time of sampling. Transcriptomics has been successfully used, for example, as a predictor of health outcomes in humans (Szabo 2014) and for the investigation of fasting metabolism in northern elephant seals (Khudyakov, Champagne, Meneghetti, & Crocker, 2017; Martinez et al., 2018). Northern elephant seal blubber transcriptome investigations facilitated the identification of many genes that were differentially expressed in response to stress, caused by acute corticosteroid elevation induced by administration of an exogenous stressor, the adrenocorticotropic hormone (ACTH) (Khudyakov et al., 2017). Additionally, in a different study, differences in expression were found in response to changes in global expression profiles before and after six to eight weeks of fasting in weaned pups (Martinez et al., 2018).

A proteomic study on blubber from harbour porpoises identified 295 different proteins; 15% of those proteins were involved in inflammation and immune response, and 11% in lipid metabolism (Kershaw, Botting, Brownlow, & Hall, 2018). It was concluded that a proteomic approach could facilitate a greater understanding of the multifunctional role of blubber Similarly, in a northern elephant seal study, metabolomic analysis described the variability in a suite of circulating metabolites that occur with fasting (Champagne et al., 2013).

Omics analyses yield huge data sets and require expert bioinformatic analysis to extract useful information. The potential of these techniques is great; however, is just starting to be explored for the evaluation of cetacean BC. The goal of omics analyses, in the context of BC biomarkers, is to identify one or several products or processes acting simultaneously that demonstrate a dependent, and ideally linear, relationship with BC. Once such candidates have been identified, their routine quantification through targeted, and cheaper approaches can be implemented.

## 6 DISCUSSION AND CONCLUSION

The powerful insight that BC estimation can provide into individual, population, and ecosystem health has made the quantification of BC desirable, and of increasing conservation importance. Although numerous methodologies have been proposed for the determination of cetacean BC, currently there is no consensus on the best approach for quantification. This review critically evaluates traditional and emerging approaches according to select criteria that encourage uptake of a measure into routine population monitoring, and comparisons between different studies, populations, and species (Table 1).

**Table 1:** Benchmarking of outlined BC approaches according to the criteria of (i) Sensitivity (S), where 3 is expected to provide a strong reflection of animal's true energy stores while 1 is expected to provide only an approximate and indirect indication. (ii) Nonlethal (NL), measure is awarded a score of 1 if it can be applied nonlethally. (iii) Cost‐efficiency (CE), on a scale of 1–3, where 3 indicates a measure that is both inexpensive to derive and easy to measure. By contrast, 1 indicates a measure that is both cumbersome and expensive to obtain. (iv) Versatility (V), is ranked on a scale of 1–5, related to suitability for application in dead as well as living animals, large as well as small species of cetaceans, immatures as well as adults and finally, suitability for pregnant females. An “*” denotes that a measure that has the potential to be applied for this category, but that species‐ and category‐specific validation and optimization remains to be performed

Approach	Measurement	Measurement variant	Idea measurement criteria									
			S	NL	CE	Versatility						Final score
						Life status		Suitable for large cetaceans	Suitable for immatures	Suitable for pregnant females	V score (out of 5)	
						Dead	Alive					
Blubber measures	Blubber mass		3	0	1	1	0	0.5	1	1	3.5	7.5
	Blubber thickness	Direct measure	1	1	1 (2 if live)	1	1	1	0	1	4	8 (7)
		Ultrasound	1	1	1	1	1	1	0.5	0	3.5	6.5
	Blubber lipid content (%)		0.5	1	3	1	1	1	0	1	4	8.5
	BTLM		3	0	1	1	0	0.5	1	1	3.5	7.5
	Adipocyte metrics	Adipocyte area	2	1	2	1	1	1	0	1	4	9
		Adipocyte index	2	1	3	1	1	1	0	1	4	10
Body composition	Carcass analysis	Bomb calorimetry	3	0	1	1	0	0.5	1	1	3.5	7.5
		Fat mass estimation	3	0	1	1	0	0.5	1	1	3.5	7.5
	Isotope dilution		2	1	1	0	1	0	1	1	3	7
	Glide pattern analysis		3	1	1	0	1	1	1	1	4	9
Body morphometry	Measurements	Body girth	2	1	2	1*	1	1	0.5*	0.5*	4	9
		Body girth‐length	2	1	2	1*	1	1	0.5*	0.5*	4	9
		UAV‐photogrammetry	3	1	2	1*	1	1	0.5*	0.5*	4	10
Chemical biomarkers	POPCI		2	1	1	1	1	1	0	1	4	8
Omics			?	1	?	1	1	1	1*	1	5	?

One of the most important criteria for any measure is sensitivity or accuracy, which is the capacity of the approach to provide a true reflection of BC, or change in the BC of a given individual or population over time. In the presented suite of approaches, the measures that may be considered the most accurate are the direct blubber measures, such as blubber mass measurements or carcass analysis, either by bomb calorimetry or by fat mass estimation. As destructive measures, these direct approaches are limited to assessment of harvested, stranded, or by‐caught animals which carries either ethical implications, or the potential to skew biological assessments. As such an inevitable compromise upon accuracy is introduced by the remaining measure criteria, that is, 2) Nonlethal, 3) Inexpensive, and 4) Versatile.

Lethal harvesting for scientific investigation not only carries ethical implications but also, it is inherently contradictory to the conservation goals of conservation research (Waugh & Monamy, 2016). Nonlethal sampling approaches range in their level of invasiveness, from live capture and release, to UAV measurements. In the middle of the spectrum are measures performed on blubber obtained through remote biopsy. Remote biopsy has gained popularity in the recent past due to the enormous amount of information that can be gathered from a single tissue sample, the significant reduction in stress to the animal, and reduction in sampling cost. This technique is now part of many field protocols all over the world, and it has been used on over 40 cetacean species (Noren & Mocklin, 2012).

The cost of acquiring a BC measure, both in terms of time and direct monetary costs, invariably plays a role in how frequently the measure is likely to be implemented. For long‐term monitoring, regular, frequent application is essential to achieve the required power of a data set to confidently ascertain temporal change in BC. Of the outlined approaches, UAV derived photogrammetry measurements, blubber histology, and biochemical and chemical biomarkers hold high potential for reduced costs, large sample throughput, and therefore routine application.

Finally, the versatility of the BC measure may influence its selection in certain research and monitoring campaigns. It is unrealistic to suggest that a single measure can be accurately applied to different species, age categories, animals of varying reproductive state, as well as live or dead individuals, without prior category‐specific validation. Indeed, a thoroughly validated measure, applied to a well‐defined group of animals, may serve its purpose for most studies. Timely advancement of the field would, however, greatly benefit from robust cross‐category testing of measures in order to ascertain measure‐specific restrictions and limitations, which in turn would facilitate faster identification of optimal measures for new research agendas.

In Table 1 the above‐discussed approaches are evaluated against the predetermined criteria rubric. The three vastly different approaches that performed the best, according to criteria rubric, were glide pattern analysis, the Adipocyte metrics  and a BC index derived using UAV‐photogrammetry, all accumulating a score of ≥ 9 out of a possible score of 12.

The key advantage that glide pattern analysis presents over other measures, aside from destructive whole‐body analyses and the POP Concentration Index, is that the measure reflects whole‐body fat stores, including visceral stores. Glide pattern analysis therefore shows good potential, although remains in the developmental stage and requires further validation, particularly with respect to shallow pitch and shallow‐water dives. As tagging of an animal is necessary, the cost‐efficiency of the approach also limits its broader monitoring application at present.

The Adipocyte Index is derived from outer blubber tissue. Blubber is one of the most readily accessible tissues, via both necropsy and remote biopsy, from which information regarding cetacean energy reserves can be derived. Nevertheless, blubber is a complex tissue with diverse and overlapping roles. The major limitation of outer blubber derived measures relate to the Outer Blubber Layer Threshold effect discussed throughout this review.

UAVs photogrammetry is based on morphological measurements applied for the calculation of BC indices. The reduction in size and cost of UAV technology has fostered rapid developments in the field, and wide‐spread application. The greatest limitations of this approach come from the index calculations, which have been the subject of other reviews, for example, (Labocha & Hayes, 2012; Labocha et al., 2014; Peig & Green, 2010; Schulte‐Hostedde et al., 2005; Wilder, Raubenheimer, & Simpson, 2016). Briefly, the standardization of the measurement across studies remains an identified need, as does the minimization and quantification of errors. Further, in standardizing and optimizing the representativeness of UAV derived BC indices, it is paramount to take into account non‐BC parameters that influence body volume, such as sex, season, and pregnancy (Boyd et al., 2010).

In addition to these three selected, well‐performing applications, continued investment into method development, with a particular focus on new technologies and interdisciplinary transfer of methods, has the potential to reduce current limitations for the benefit of diverse areas of cetacean research and monitoring.

## 7 IDENTIFIED PRIORITIES

The key research priorities identified through this review can be summarized as follow:

- Implementation of diverse measures in parallel on individuals and populations representative of a spectrum of BC states is needed. This would be of benefit for both closely related measures, such as blubber thickness and blubber lipid content, as well as vastly different measures, such as glide dynamics and AI. Such quality assurance studies would serve to highlight the strengths and weaknesses of, and therefore optimal application for, each measure.
- Continued development, validation, and streamlining of leading applications. For example, ancillary data regarding pregnancy, as determined through blubber steroid hormone analysis, would provide a sample set for exploration and testing of how pregnancy is likely to influence BC quantification via 3D modeling techniques, and ultimately how the error might be managed.
- Further investment into research and development for the identification of new chemical and biochemical markers of BC. Uptake of new technological advances into cetacean applications holds vast potential for advancement of the field.
- Finally, the above priority simultaneously holds excellent potential for advancement of our understanding of blubber and its complex role in cetacean homeostasis.

## AUTHOR CONTRIBUTION

**Juliana Castrillon:** Conceptualization (equal); Investigation (lead); Writing – original draft (lead); Writing‐review & editing (equal).**Susan Bengtson Nash:** Conceptualization (lead); Project administration (lead); Supervision (lead); Writing‐review & editing (equal).

JC conduct the literature review and manuscript draft and SBN rework manuscript for publication.

## Data Availability

This is a review paper without any original empirical data.

## References

[ece36301-bib-0001] Ackman, R. G. , Hingley, J. , Eaton, C. , Logan, V. , & Odense, P. (1975a). Layering and tissue composition in the blubber of the northwest Atlantic sei whale (Balaenoptera borealis). Canadian Journal of Zoology, 53, 1340–1344. 1203817 10.1139/z75-159

[ece36301-bib-0002] Ackman, R. G. , Hingley, J. , Eaton, C. , Sipos, J. , & Mitchell, E. (1975b). Blubber fat deposition in mysticeti whales. Canadian Journal of Zoology, 53, 1332–1339. 10.1139/z75-158 1203816

[ece36301-bib-0003] Adamczak, S. K. , Pabst, A. , McLellan, W. , & Thorne, L. (2019). Using 3D models to improve estimates of marine mammal size and external morphology. Frontiers in Marine Science, 6, 334 10.3389/fmars.2019.00334

[ece36301-bib-0004] Aguilar, A. , & Borrell, A. (1990). Patterns of lipid content and stratification in the blubber of fin whales (Balaenoptera physalus). Journal of Mammalogy, 71, 544–554. 10.2307/1381793

[ece36301-bib-0005] Aguilar, A. , Borrell, A. , & Gómez‐Campos, E. (2007). The reliability of blubber thickness as a measure of body condition in large whales. In: 59th Annual Meeting of the Scientific Committee of the International Whaling Commission (SC/59/O17)

[ece36301-bib-0006] Aguilar, A. , Borrell, A. , & Pastor, T. (1999). Biological factors affecting variability of persistent pollutant levels in cetaceans. Journal of Cetacean Research and Management, 1:83–116.

[ece36301-bib-0007] Arnould, J. P. Y. (1995). Indices of body condition and body composition in female antarctic fur seal (*Arctocephalus gazella*). Marine Mammal Science, 11, 301–313.

[ece36301-bib-0008] Atkinson, A. , Colburn, W. A. , DeGruttola, V. G. , DeMets, D. L. , Downing, G. J. , Hoth, D. F. , … Spilker, B. A. (2001). Biomarkers and surrogate endpoints: Preferred definitions and conceptual framework. Clinical Pharmacology and Therapeutics, 69, 89–95. 11240971 10.1067/mcp.2001.113989

[ece36301-bib-0009] Ball, H. , Londraville, R. , Prokop, J. , George, J. C. , Suydam, R. , Vinyard, C. , … Duff, R. (2017). Beyond thermoregulation: Metabolic function of cetacean blubber in migrating bowhead and beluga whales. Journal of Comparative Physiology B, 187, 235–252. 10.1007/s00360-016-1029-6 PMC5535305 27573204

[ece36301-bib-0010] Ball, H. C. , Stavarz, M. , Oldaker, J. , Usip, S. , Londraville, R. L. , George, J. C. , … Duff, R. J. (2015). Seasonal and ontogenetic variation in subcutaneous adipose of the bowhead whale (Balaena mysticetus). The Anatomical Record, 298, 1416–1423. 25711480 10.1002/ar.23125

[ece36301-bib-0011] Bateson, P. (1986). When to experiment on animals. New Scientist, 109, 30–32. 11655736

[ece36301-bib-0012] Beck, G. G. , Smith, T. G. , & Hammill, M. O. (1993). Evaluation of body condition in the northwest atlantic harp seal (Phoca groenlandica). Canadian Journal of Fisheries and Aquatic Sciences, 50, 1372–1381.

[ece36301-bib-0013] Bell, C. M. , Hindell, M. A. , & Burton, H. R. (1997). Estimation of body mass in the southern elephant seal, Mirounga leonina, by photogrammetry and morphometrics. Marine Mammal Science, 13, 669–682. 10.1111/j.1748-7692.1997.tb00090.x

[ece36301-bib-0014] Bengtson Nash, S. (2018a). Toxicological risks and considerations associated with lipophilic contaminant burdens of southern ocean mysticetes In: Fossi C. & Panti C. (Eds.), Marine Mammal Ecotoxicology: impacts of multiple stressors on population health (pp. 381‐399). UK: Elsevier.

[ece36301-bib-0015] Bengtson Nash, S. M. (2018b). Toxicological risks and considerations associated with lipophilic contaminant burdens of Southern Ocean Mysticetes Marine mammal ecotoxicology (pp. 381–400). Elsevier.

[ece36301-bib-0016] Bengtson Nash, S. M. , Castrillon, J. , Eisenmann, P. , Fry, B. , Shuker, J. D. , Cropp, R. A. , … McLagan, D. (2017). Signals from the south; humpback whales carry messages of Antarctic sea‐ice ecosystem variability. Global Change Biology, 24, 1500–1510. 10.1111/gcb.14035 29284198

[ece36301-bib-0017] Bengtson Nash, S. M. , Waugh, C. A. , & Schlabach, M. (2013a). Metabolic Concentration of Lipid Soluble Organochlorine Burdens in Humpback Whales Through Migration and Fasting. Environmental Science and Technology, 47, 9404–9413. 23859482 10.1021/es401441n

[ece36301-bib-0018] Bengtson Nash, S. M. , Waugh, C. A. , & Schlabach, M. (2013b). Metabolic concentration of lipid soluble organochlorine burdens in the blubber of southern hemisphere humpback whales through migration and fasting. Environmental Science & Technology, 47, 9404–9413. 10.1021/es401441n 23859482

[ece36301-bib-0019] Best, P. , & Rüther, H. (1992). Aerial photogrammetry of southern right whales, Eubalaena australis. Journal of Zoology, 228, 595–614.

[ece36301-bib-0020] Biuw, M. , McConnell, B. , Bradshaw, C. J. A. , Burton, H. , & Fedak, M. (2003). Blubber and buoyancy: Monitoring the body condition of free‐ranging seals using simple dive characteristics. Journal of Experimental Biology, 206, 3405–3423. 10.1242/jeb.00583 12939372

[ece36301-bib-0021] Bligh, E. G. , & Dyer, W. J. (1959). A rapid method of total lipid extraction and purification. Canadian Journal of Biochemistry and Physiology, 37, 911–917. 10.1139/o59-099 13671378

[ece36301-bib-0022] Bowen, W. D. , & Iverson, S. J. (1998). Estimation of total body water in pinnipeds using hydrogen‐isotope dilution. Physiological Zoology, 71, 329–332. 10.1086/515921 9634180

[ece36301-bib-0023] Boyd, I. L. , Bowen, D. W. , & Iverson, S. (2010). Marine mammal ecology and conservation: A handbook of Techniques. Oxford: Oxford University Press.

[ece36301-bib-0024] Bräger, S. , & Chong, A. (1999). An application of close range photogrammetry in dolphin studies. The Photogrammetric Record, 16, 503–517. 10.1111/0031-868X.00139

[ece36301-bib-0025] Brager, S. , Chong, A. , Dawson, S. , Slooten, E. , & Wursig, B. (1999). A combined stereo‐photogrammetry and underwater‐video system to study group composition of dolphins. Helgoland Marine Research, 53, 122–128. 10.1007/s101520050015

[ece36301-bib-0026] Braithwaite, J. , Meeuwig, J. , Letessier, T. , Jenner, K. C. , & Brierley, A. (2015). From sea ice to blubber: Linking whale condition to krill abundance using historical whaling records. Polar Biology, 38: 1–8. 10.1007/s00300-015-1685-0

[ece36301-bib-0027] Budge, S. M. , Iverson, S. J. , & Koopman, H. N. (2006). Studying trophic ecology in marine ecosystems using fatty acids: A primer on analysis and interpretation. Marine Mammal Science, 22, 759–801. 10.1111/j.1748-7692.2006.00079.x

[ece36301-bib-0028] Burnett, J. D. , Lemos, L. , Barlow, D. , Wing, M. G. , Chandler, T. , & Torres, L. G. (2018). Estimating morphometric attributes of baleen whales with photogrammetry from small UASs: A case study with blue and gray whales. Marine Mammal Science, 35(1), 108–139

[ece36301-bib-0029] Caon, G. , Fialho, C. B. , & Danilewicz, D. (2007). Body fat condition in franciscanas (Pontoporia blainvillei) in Rio Grande do Sul, southern Brazil. Journal of Mammalogy, 88, 1335–1341. 10.1644/06-MAMM-A-364R.1

[ece36301-bib-0030] Cartee, R. , Gray, B. , John, J. , & Ridgway, S. (1995). B‐mode ultrasound evaluation of dolphin skin. Journal of Diagnostic Medical Sonography, 11, 76–80. 10.1177/875647939501100205

[ece36301-bib-0031] Casa, V. , Dalle, Luche G. , Ghosh, R. , Bohlin‐Nizzetto, P. , Burkard, M. , Schirmer, K. , & Bengtson, Nash S. (2019) Emerging contaminants in humpback whales (Megaptera novaeangliae) foraging in Antarctic waters, and their impact on whale fibroblast cell viability. In: World Marine Mammal Conference, Barcelona, Spain.

[ece36301-bib-0032] Castellini, M. A. , & Mellish, J.‐A. (2015). Marine Mammal Physiology: Requisites for Ocean Living. Boca Raton: CRC Press.

[ece36301-bib-0033] Castellini, M. A. , & Rea, L. D. (1992). The Biochemistry of Natural Fasting at Its Limits., 48, 575–582. 10.1007/BF01920242 1612138

[ece36301-bib-0034] Castrillon, J. , Huston, W. , & Bengtson Nash, S. (2017) The blubber adipocyte index: A nondestructive biomarker of adiposity in humpback whales (Megaptera novaeangliae). Ecology and Evolution, 7(14): 5131‐5139. 28770053 10.1002/ece3.2913 PMC5528216

[ece36301-bib-0035] Cattet, M. R. , Atkinson, S. N. , Polischuk, S. C. , & Ramsay, M. A. (1997). Predicting body mass in polar bears: Is morphometry useful? The Journal of Wildlife Management, 61, 1083–1090. 10.2307/3802105

[ece36301-bib-0036] Champagne, C. D. , Boaz, S. M. , Fowler, M. A. , Houser, D. S. , Costa, D. P. , & Crocker, D. E. (2013). A profile of carbohydrate metabolites in the fasting northern elephant seal. Comparative Biochemistry and Physiology Part D: Genomics and Proteomics, 8, 141–151. 10.1016/j.cbd.2013.02.002 23542762

[ece36301-bib-0037] Christiansen, F. , Dujon, A. M. , Sprogis, K. R. , Arnould, J. P. Y. , & Bejder, L. (2016). Noninvasive unmanned aerial vehicle provides estimates of the energetic cost of reproduction in humpback whales. Ecosphere, 7, e01468, n/a. 10.1002/ecs2.1468

[ece36301-bib-0038] Christiansen, F. , Sprogis, K. R. , Gross, J. , Castrillon, J. , Warick, H. A. , Leunissen, E. , & Bengtson Nash, S. (2020) Variation in outer blubber lipid concentrations does not reflect morphological body condition in humpback whales. Journal of Experimental Biology, 233(8): jeb213769. 10.1242/jeb.213769 32165431

[ece36301-bib-0039] Christiansen, F. , Víkingsson, G. A. , Rasmussen, M. H. , & Lusseau, D. (2013). Minke whales maximise energy storage on their feeding grounds. The Journal of Experimental Biology, 216, 427–436. 10.1242/jeb.074518 23325860

[ece36301-bib-0040] Christiansen, F. , Víkingsson, G. A. , Rasmussen, M. H. , & Lusseau, D. (2014). Female body condition affects foetal growth in a capital breeding mysticete. Functional Ecology, 28, 579–588. 10.1111/1365-2435.12200

[ece36301-bib-0041] Christiansen, F. , Vivier, F. , Charlton, C. , Ward, R. , Amerson, A. , Burnell, S. , & Bejder, L. (2018). Maternal body size and condition determine calf growth rates in southern right whales. Marine Ecology Progress Series, 592, 267–281. 10.3354/meps12522

[ece36301-bib-0042] Clapham, P. J. , & Ivashchenko, Y. V. (2018). Whaling catch data are not reliable for analyses of body size shifts. Nature Ecology & Evolution, 1, 756–756. 10.1038/s41559-018-0534-2 29662227

[ece36301-bib-0043] Clarke, R. H. , Aguayo, L. A. , & Obla, P. G. (1972). Sperm Whales of the Southeast Pacific: By Robert Clarke. Copenhagen: Universitetsforlaget.Christiansen

[ece36301-bib-0044] Cook, R. C. , Cook, J. G. , Murray, D. L. , Zager, P. , Johnson, B. K. , & Gratson, M. W. (2001). Nutritional condition models for elk: which are the most sensitive, accurate, and precise? The Journal of Wildlife Management, 65, 988–997. 10.2307/3803047

[ece36301-bib-0045] Cornick, L. A. , Quakenbush, L. T. , Norman, S. A. , Pasi, C. , Maslyk, P. , Burek, K. A. , … Hobbs, R. C. (2016). Seasonal and developmental differences in blubber stores of beluga whales in Bristol Bay, Alaska using high‐resolution ultrasound. Journal of Mammalogy, 97(4), 1238–1248. 10.1093/jmammal/gyw074 29899579 PMC5993092

[ece36301-bib-0046] Cropp, R. , Bengtson Nash, S. M. , & Hawker, D. (2014). A model to resolve the dynamics of organochlorine pharmacokinetics in migrating humpback whales. Environmental Toxicology and Chemistry, 33, 1638–1649. 24733631 10.1002/etc.2603

[ece36301-bib-0047] Cubbage, J. C. , & Calambokidis, J. (1987). Size‐class segregation of bowhead whales discerned through aerial stereophotogrammetry. Marine Mammal Science, 3, 179–185. 10.1111/j.1748-7692.1987.tb00160.x

[ece36301-bib-0048] Curran, M. P. , & Asher, W. M. (1974). Investigation of blubber thickness in a gray whale using ultrasonography. Marine Fisheries Review, 36, 15–20.

[ece36301-bib-0049] Druskat, A. , Ghosh, R. , Castrillon, J. , & Bengtson Nash, S. M. (2019). Sex ratios of migrating southern hemisphere humpback whales: A new sentinel parameter of ecosystem health. Marine Environmental Research, 151, 104749 10.1016/j.marenvres.2019.104749 31256980

[ece36301-bib-0050] Dunkin, R. C. , McLellan, W. A. , Blum, J. E. , & Pabst, D. A. (2005). The ontogenetic changes in the thermal properties of blubber from Atlantic bottlenose dolphin (Tursiops truncatus). Journal of Experimental Biology, 208, 1469–1480. 10.1242/jeb.01559 15802671

[ece36301-bib-0051] Durban, J. W. , Fearnbach, H. , Barrett‐Lennard, L. G. , Perryman, W. L. , & Leroi, D. J. (2015). Photogrammetry of killer whales using a small hexacopter launched at sea. Journal of Unmanned Vehicle Systems, 3, 131–135. 10.1139/juvs-2015-0020

[ece36301-bib-0052] Durban, J. , & Parsons, K. (2006). Laser‐metrics of free‐ranging killer whales. Marine Mammal Science, 22, 735–743. 10.1111/j.1748-7692.2006.00068.x

[ece36301-bib-0053] Elfes, C. T. (2008). Persistent organic pollutant levels in North Pacific and North Atlantic humpback whales (Megaptera novaeangliae) School of Aquatic and Fishery Sciences (p. 98). University of Washington. PhD Thesis

[ece36301-bib-0054] Evans, K. , Hindell, M. A. , & Thiele, D. (2003). Body fat and condition in sperm whales, Physeter macrocephalus, from southern Australian waters. Comparative Biochemistry and Physiology Part A: Molecular & Integrative Physiology, 134, 847–862. 10.1016/S1095-6433(03)00045-X 12814793

[ece36301-bib-0055] Faust, I. M. , Johnson, P. R. , Stern, J. S. , & Hirsch, J. (1978). Diet‐induced adipocyte number increase in adult rats: A new model of obesity. American Journal of Physiology ‐ Endocrinology and Metabolism, 235, E279 10.1152/ajpendo.1978.235.3.E279 696822

[ece36301-bib-0056] Fish, F. E. (2000). Biomechanics and energetics in aquatic and semiaquatic mammals: Platypus to whale. Physiological and Biochemical Zoology, 73, 683–698. 10.1086/318108 11121343

[ece36301-bib-0057] Folch, J. , Lees, M. , & Sloane‐Stanley, G. (1957). A simple method for the isolation and purification of total lipids from animal tissues. Journal of Biological Chemistry, 226, 497–509. 13428781

[ece36301-bib-0058] Forsyth, D. M. , Duncan, R. P. , Tustin, K. G. , & Gaillard, J.‐M. (2005). A substantial energetic cost to male reproduction in a sexually dimorphic ungulate. Ecology, 86, 2154–2163. 10.1890/03-0738

[ece36301-bib-0059] Frid , & Dill, L. (2002). Human‐caused disturbance stimuli as a form of predation risk. Conservation Ecology, 6(1).

[ece36301-bib-0060] Gales, N. , & Burton, H. (1987). Ultrasonic measurement of blubber thickness of the southern elephant seal, mirounga‐leonina (Linn). Australian Journal of Zoology, 35, 207–217. 10.1071/ZO9870207

[ece36301-bib-0061] George, J. C. , Druckenmiller, M. L. , Laidre, K. L. , Suydam, R. , & Person, B. (2015). Bowhead whale body condition and links to summer sea ice and upwelling in the Beaufort Sea. Progress in Oceanography, 136, 250–262. 10.1016/j.pocean.2015.05.001

[ece36301-bib-0062] Gómez‐Campos, E. , Borrell, A. , & Aguilar, A. (2011). Assessment of nutritional condition indices across reproductive states in the striped dolphin (Stenella coeruleoalba). Journal of Experimental Marine Biology and Ecology, 405, 18–24. 10.1016/j.jembe.2011.05.013

[ece36301-bib-0063] Gosler, A. G. (1996). Environmental and social determinants of winter fat storage in the great tit Parus major. Journal of Animal Ecology, 65, 1–17. 10.2307/5695

[ece36301-bib-0064] Green, A. J. (2001). Mass/leng residuals: Measures of body consition or generators of spurious results? Ecology, 82, 1473–1483.

[ece36301-bib-0065] Hall, K. D. , Heymsfield, S. B. , Kemnitz, J. W. , Klein, S. , Schoeller, D. A. , & Speakman, J. R. (2012). Energy balance and its components: Implications for body weight regulation. The American Journal of Clinical Nutrition, 95, 989–994. 10.3945/ajcn.112.036350 22434603 PMC3302369

[ece36301-bib-0066] Hanks, J. (1981). Characterisation of population condition In Fowler C. & Smith T. (Eds.), Dynamics of large mammal populations (pp. 47–73). Wiley: New York.

[ece36301-bib-0067] Harwood, L. , Smith, T. , George, J. , Sandstrom, S. , Walkusz, W. , & Divoky, G. (2015). Change in the Beaufort Sea ecosystem: Diverging trends in body condition and/or production in five marine vertebrate species. Progress in Oceanography, 136, 263–273. 10.1016/j.pocean.2015.05.003

[ece36301-bib-0068] Haug, T. , Lindstrøm, U. , & Nilssen, K. T. (2002). Variations in minke whale (Balaenoptera acutorostrata) diet and body condition in response to ecosystem changes in the barents sea. Sarsia, 87, 409–422.

[ece36301-bib-0069] Horgan, R. P. , & Kenny, L. C. (2011). ‘Omic’ technologies: Genomics, transcriptomics, proteomics and metabolomics. The Obstetrician & Gynaecologist, 13, 189–195.

[ece36301-bib-0070] Hui, C. A. (1981). Seawater consumption and water flux in the common dolphin Delphinus delphis. Physiological Zoology, 54, 430–440. 10.1086/physzool.54.4.30155836

[ece36301-bib-0071] Hunt, K. E. , Moore, M. J. , Rolland, R. M. , Kellar, N. M. , Hall, A. J. , Kershaw, J. , … Kraus, S. D. (2013). Overcoming the challenges of studying conservation physiology in large whales: A review of available methods. Conservation. Physiology, 1, cot006–cot006. 10.1093/conphys/cot006 PMC4806609 27293590

[ece36301-bib-0072] Ichii, T. , Shinohara, N. , Fujise, Y. , Nishiwaki, S. , & Matsuoka, K. (1998). Interannual changes in body fat condition index of minke whales in the Antarctic. Marine Ecology Progress Series, 175, 1–12. 10.3354/meps175001

[ece36301-bib-0073] Irvine, L. G. , Thums, M. , Hanson, C. E. , McMahon, C. R. , & Hindell, M. A. (2017). Quantifying the energy stores of capital breeding humpback whales and income breeding sperm whales using historical whaling records. Royal Society Open Science, 4, 160290 10.1098/rsos.160290 28405350 PMC5383807

[ece36301-bib-0074] Iverson, S. (2002). Blubber In Würsig B., & Thewissen J. G. M., &Perrin W. F. (Eds.), Encyclopedia of marine mammals (pp. 107–112) San Diego: Academic Press San Diego (USA).

[ece36301-bib-0075] Iverson, S. , & Koopman, H. N. (2018). Blubber In Würsig B., Thewissen J., & Kovacs K. M. (Eds.), Encyclopedia of marine mammals, San Diego: Academic Press.

[ece36301-bib-0076] Iverson, S. J. , Sparling, C. E. , Williams, T. M. , & Shelley, L. (2010). Measurement of individual and population energetics of marine mammals (pp. 165–189). In Boyd, Bowen, & Iversen(Eds,). Marine mammal ecology and conservation. Oxford: Oxfors University Press.

[ece36301-bib-0077] IWC (2001). Report of the workshop on status and trends of western North Atlantic right whales. Journal of Cetacean Research and Management, 2, 61–87.

[ece36301-bib-0078] Jakob, E. M. , Marshall, S. D. , & Uetz, G. W. (1996). Estimating fitness: A comparison of body condition indices. Oikos, 77, 61–67. 10.2307/3545585

[ece36301-bib-0079] Jaquet, N. (2006). A simple photogrammetric technique to measure sperm whales at sea. Marine Mammal Science, 22, 862–879. 10.1111/j.1748-7692.2006.00060.x

[ece36301-bib-0080] Kastelein, R. , Van der Sijs, S. , Staal, C. , & Nieuwstraten, S. (1995) Blubber thickness in harbour porpoises (Phocoena phocoena). Food consumption and growth of marine mammals In Read A.J., Wiepkema P. R., & Nachtigall P. E(Eds.), The biology of the harbour porpoise(pp. 179‐199). Woerden, Netherlands: De Spil Publishers.

[ece36301-bib-0081] Kershaw, J. L. , Botting, C. H. , Brownlow, A. , & Hall, A. J. (2018). Not just fat: Investigating the proteome of cetacean blubber tissue. Conservation Physiology, 6:1‐15. 10.1093/conphys/coy003 PMC5814904 29479430

[ece36301-bib-0082] Kershaw, J. L. , Brownlow, A. , Ramp, C. A. , Miller, P. J. O. , & Hall, A. J. (2019). Assessing cetacean body condition: Is total lipid content in blubber biopsies a useful monitoring tool? Aquatic Conservation: Marine and Freshwater Ecosystems, 29, 271–282. 10.1002/aqc.3105

[ece36301-bib-0083] Kershaw, J. L. , Sherrill, M. , Davison, N. J. , Brownlow, A. , & Hall, A. J. (2017). Evaluating morphometric and metabolic markers of body condition in a small cetacean, the harbour porpoise (Phocoena phocoena). Ecology and Evolution, 7, 3494–3506. 28515885 10.1002/ece3.2891 PMC5433969

[ece36301-bib-0084] Khudyakov, J. I. , Champagne, C. D. , Meneghetti, L. M. , & Crocker, D. E. (2017). Blubber transcriptome response to acute stress axis activation involves transient changes in adipogenesis and lipolysis in a fasting‐adapted marine mammal. Scientific Reports, 7, 42110 10.1038/srep42110 28186107 PMC5301240

[ece36301-bib-0085] Konishi, K. (2006). Characteristics of blubber distribution and body condition indicators for Antarctic minke whales (Balaenoptera bonaerensis). Mammal Study, 31, 15–22. 10.3106/1348-6160(2006)31[15:COBDAB]2.0.CO;2

[ece36301-bib-0086] Konishi, K. , Tamura, T. , Zenitani, R. , Bando, T. , Kato, H. , & Walløe, L. (2008). Decline in energy storage in the Antarctic minke whale (Balaenoptera bonaerensis) in the Southern Ocean. Polar Biology, 31, 1509–1520. 10.1007/s00300-008-0491-3

[ece36301-bib-0087] Koopman, H. N. (2007). Phylogenetic, ecological, and ontogenetic factors influencing the biochemical structure of the blubber of odontocetes. Marine Biology, 151, 277–291. 10.1007/s00227-006-0489-8

[ece36301-bib-0088] Koopman, H. , Iverson, S. , & Gaskin, D. (1996a). Stratification and age‐related differences in blubber fatty acids of the male harbour porpoise (Phocoena phocoena). Journal of Comparative Physiology B, 165, 628–639. 10.1007/BF00301131 8882509

[ece36301-bib-0089] Koopman, H. N. , Pabst, D. A. , McLellan, W. A. , Dillaman, R. M. , & Read, A. J. (2002). Changes in blubber distribution and morphology associated with starvation in the harbour porpoise (Phocoena Phocoena): Evidence for regional differences in blubber structure and function. Physiological and Biochemical Zoology, 75, 498–512. 12529851 10.1086/342799

[ece36301-bib-0090] Koski, W. , Rugh, D. , Punt, A. , & Zeh, J. (2006). An approach to minimise bias in estimation of the length‐frequency distribution of bowhead whales (Balaena mysticetus) from aerial photogrammetric data. Journal of Cetacean Research and Management, 8, 45.

[ece36301-bib-0091] Krahn, M. M. , Herman, D. P. , Ylitalo, G. M. , Sloan, C. A. , Burrows, D. G. , Hobbs, R. C. , … Moore, S. E. (2004b). Stratification of lipids, fatty acids and organochlorine contaminants in blubber of white whales and killer whales. Journal of Cetacean Research and Management, 6, 175–189.

[ece36301-bib-0092] Krahn, M. M. , Herman, D. P. , Ylitalo, G. M. , Sloan, C. A. , Burrows, D. G. , Hobbs, R. C. , … Moore, S. (2004a). Stratification of lipids, fatty acids and organochlorine contaminants in blubber of white whales and killer whales. Journal of Cetacean Research and Management, 6, 175–189.

[ece36301-bib-0093] Krahn, M. M. , Ylitalo, G. M. , Burrows, D. G. , Calambokidis, J. , Moore, S. E. , Gosho, M. , … Blokhin, S. (2001). Organochlorine contaminant concentrations and lipid profiles in eastern North Pacific gray whales(Eschrichtius robustus). Journal of Cetacean Research and Management, 3, 19–29.

[ece36301-bib-0094] Krebs, C. J. , & Singleton, G. R. (1993). Indexes of condition for small mammals. Australian Journal of Zoology, 41, 317–323. 10.1071/ZO9930317

[ece36301-bib-0095] Kvadsheim, P. , Folkow, L. , & Blix, A. (1996). Thermal conductivity of minke whale blubber. Journal of Thermal Biology, 21, 123–128. 10.1016/0306-4565(95)00034-8

[ece36301-bib-0096] Labocha, M. K. , & Hayes, J. P. (2012). Morphometric indices of body condition in birds: A review. Journal of Ornithology, 153, 1–22. 10.1007/s10336-011-0706-1

[ece36301-bib-0097] Labocha, M. K. , Schutz, H. , & Hayes, J. P. (2014). Which body condition index is best? Oikos, 123, 111–119. 10.1111/j.1600-0706.2013.00755.x

[ece36301-bib-0098] Lane, J. E. , Boutin, S. , Speakman, J. R. , & Humphries, M. M. (2010). Energetic costs of male reproduction in a scramble competition mating system. Journal of Animal Ecology, 79, 27–34. 10.1111/j.1365-2656.2009.01592.x 19674182

[ece36301-bib-0099] Lockyer, C. (1976). Body weights of some species of large whales. Journal Du Conseil, 36, 259–273. 10.1093/icesjms/36.3.259

[ece36301-bib-0100] Lockyer, C. (1986). Body fat condition in northeast Atlantic fin whales, Balaenoptera Physalus, and its relationship with reproduction and food resource. Canadian Journal of Fisheries and Aquatic Sciences, 43, 142–147.

[ece36301-bib-0101] Lockyer, C. (1987a). Evaluation of the role of fat reserves in relation to the ecology of North Atlantic fin and sei whales . In Huntley A.C., Costa D. P., Worthy G. A. J., & Castellini M. A. (Eds.), Approaches to marine mammal energetics(pp. 183–203). Society of Marine Mammalogy.

[ece36301-bib-0102] Lockyer, C. (1987b) The relationship between body fat, food resource and reproductive energy costs in north Atlantic fin whales (Balaenoptera physalus) In: Symposia of the Zoological Society of London, (pp. 343–361). Symp. Zool. Soc. Lond: Published for the Zoological Society by Academic Press.

[ece36301-bib-0103] Lockyer, C. (1991). Body composition of the sperm whale, Physeter catodon, with special reference to the possible functions of fat depots. Marine Research Institute.

[ece36301-bib-0104] Lockyer, C. (2007a). All creatures great and smaller: A study in cetacean life history energetics. Journal of the Marine Biological Association of the United Kingdom, 87, 1035–1045. 10.1017/S0025315407054720

[ece36301-bib-0105] Lockyer, C. H. , McConnell, L. C. , & Waters, T. D. (1984). The biochemical composition of fin whale blubber. Canadian Journal of Zoology, 62, 2553–2562. 10.1139/z84-373

[ece36301-bib-0106] Lockyer, C. , McConnell, L. , & Waters, T. (1985a). Body condition in terms of anatomical and biochemical assessment of body fat in North Atlantic fin and sei whales. Canadian Journal of Zoology, 63, 2328–2338. 10.1139/z85-345

[ece36301-bib-0107] Lockyer, C. , & Waters, T. (1986). Weights and anatomical measurements of northeastern Atlantic fin (Balaenoptera physalus, Linnaeus) and sei (B. borealis, Lesson) whales. Marine Mammal Science, 2, 169–185. 10.1111/j.1748-7692.1986.tb00039.x

[ece36301-bib-0108] Luque, S. P. , & Aurioles‐Gamboa, D. (2001). Sex differences in body size and body condition of California sea lion (Zalophus californianus) pups from the Gulf of California. Marine Mammal Science, 17, 147–160. 10.1111/j.1748-7692.2001.tb00985.x

[ece36301-bib-0109] Martinez, B. , Khudyakov, J. , Rutherford, K. , Crocker, D. E. , Gemmell, N. , & Ortiz, R. M. (2018). Adipose transcriptome analysis provides novel insights into molecular regulation of prolonged fasting in northern elephant seal pups. Physiological Genomics, 50, 495–503. 10.1152/physiolgenomics.00002.2018 29625017 PMC6087879

[ece36301-bib-0110] McKinney, M. A. , Atwood, T. , Dietz, R. , Sonne, C. , Iverson, S. J. , & Peacock, E. (2014). Validation of adipose lipid content as a body condition index for polar bears. Ecology and Evolution, 4, 516–527. 10.1002/ece3.956 24634735 PMC3936397

[ece36301-bib-0111] McLellan, W. A. , Koopman, H. N. , Rommel, S. A. , Read, A. J. , Potter, C. W. , Nicolas, J. R. , … Pabst, D. A. (2002). Ontogenetic allometry and body composition of harbour porpoises (Phocoena phocoena, L.) from the western North Atlantic. Journal of Zoology, 257, 457–471. 10.1017/S0952836902001061

[ece36301-bib-0112] McMahon, C. R. , Harcourt, R. , Bateson, P. , & Hindell, M. A. (2012). Animal welfare and decision making in wildlife research. Biological Conservation, 153, 254–256. 10.1016/j.biocon.2012.05.004

[ece36301-bib-0113] Miller, C. A. , Best, P. B. , Perryman, W. L. , Baumgartner, M. F. , & Moore, M. J. (2012). Body shape changes associated with reproductive status, nutritive condition and growth in right whales Eubalaena glacialis and E. australis. Marine Ecology Progress Series, 459, 135–156. 10.3354/meps09675

[ece36301-bib-0114] Miller, C. A. , Reeb, D. , Best, P. B. , Knowlton, A. R. , Brown, M. W. , & Moore, M. J. (2011). Blubber thickness in right whales Eubalaena glacialis and Eubalaena australis related with reproduction, life history status and prey abundance. Marine Ecology Progress Series, 438, 267–283. 10.3354/meps09174

[ece36301-bib-0115] Miller, P. , & Hall, A. (2012). Behavioral Ecology of Cetaceans: The Relationship of Body Condition with Behavior and Reproductive Success. DTIC Document.

[ece36301-bib-0116] Miller, P. J. O. , Johnson, M. P. , Tyack, P. L. , & Terray, E. A. (2004). Swimming gaits, passive drag and buoyancy of diving sperm whales (Physeter macrocephalus). Journal of Experimental Biology, 207, 1953–1967. 10.1242/jeb.00993 15107448

[ece36301-bib-0117] Miller, P. , Narazaki, T. , Isojunno, S. , Aoki, K. , Smout, S. , & Sato, K. (2016). Body density and diving gas volume of the northern bottlenose whale (Hyperoodon ampullatus). Journal of Experimental Biology, 219, 2458–2468. 27296044 10.1242/jeb.137349 PMC5004977

[ece36301-bib-0118] Montie, E. W. , Garvin, S. R. , Fair, P. A. , Bossart, G. D. , Mitchum, G. B. , McFee, W. E. , … Hahn, M. E. (2008). Blubber morphology in wild bottlenose dolphins (Tursiops truncatus) from the Southeastern United States: Influence of geographic location, age class, and reproductive state. Journal of Morphology, 269, 496–511. 10.1002/jmor.10602 18157858

[ece36301-bib-0119] Moore, M. , Miller, C. , Morss, M. , Arthur, R. , Lange, W. , Prada, K. , … Frey, E. (2001). Ultrasonic measurement of blubber thickness in right whales. Journal of Cetacean Research and Management (Special Issue), 2, 301–309.

[ece36301-bib-0120] Narazaki, T. , Isojunno, S. , Nowacek, D. P. , Swift, R. , Friedlaender, A. S. , Ramp, C. , … Miller, P. J. O. (2018). Body density of humpback whales (Megaptera novaengliae) in feeding aggregations estimated from hydrodynamic gliding performance. PLoS ONE, 13, e0200287 10.1371/journal.pone.0200287 30001369 PMC6042725

[ece36301-bib-0121] Niæss, A. , Haug, T. , & Nilssen, E. M. (1998). Seasonal variation in body condition and muscular lipid contents in northeast atlantic minke whale, Balaenoptera acutorostrata. Sarsia, 83, 211–218.

[ece36301-bib-0122] Nolan, C. , & Liddle, G. (2000). Measuring size of humpback whales Megaptera novaeangliae by underwater videogrammetry. Marine Mammal Science, 16, 664–676.

[ece36301-bib-0123] Noren, D. P. , & Mocklin, J. A. (2012). Review of cetacean biopsy techniques: Factors contributing to successful sample collection and physiological and behavioral impacts. Marine Mammal Science, 28, 154–199. 10.1111/j.1748-7692.2011.00469.x

[ece36301-bib-0124] Noren, S. R. , Udevitz, M. S. , Triggs, L. , Paschke, J. , Oland, L. , & Jay, C. V. (2015). Identifying a reliable blubber measurement site to assess body condition in a marine mammal with topographically variable blubber, the Pacific walrus. Marine Mammal Science, 31, 658–676. 10.1111/mms.12186

[ece36301-bib-0125] Olsen, E. , & Grahl‐Nielsen, O. (2003). Blubber fatty acids of minke whales: Stratification, population identification and relation to diet. Marine Biology, 142, 13–24. 10.1007/s00227-002-0934-2

[ece36301-bib-0126] Pace, N. , & Rathbun, E. N. (1945). Studies on body composition. 3. The body water and chemically combined nitrogen content in relation to fat content. Journal of Biological Chemistry, 158, 685–691.

[ece36301-bib-0127] Parry, D. (1949). The structure of whale blubber, and a discussion of its thermal properties. Quarterly Journal of Microscopical Science, 3, 13–25. 18128472

[ece36301-bib-0128] Peig, J. , & Green, A. J. (2010). The paradigm of body condition: A critical reappraisal of current methods based on mass and length. Functional Ecology, 24, 1323–1332. 10.1111/j.1365-2435.2010.01751.x

[ece36301-bib-0129] Perryman, W. L. , & Lynn, M. S. (2002). Evaluation of nutritive condition and reproductive status of migrating gray whales (Eschrichtius robustus) based on analysis of photogrammetric data. Journal of Cetacean Research and Management, 4, 155–164.

[ece36301-bib-0130] Read, A. J. (1990). Estimation of body condition in harbour porpoises, Phocoena phocoena. Canadian Journal of Zoology, 68, 1962–1966.

[ece36301-bib-0131] Reilly, J. J. , & Fedak, M. (1990). Measurement of the body composition of living gray seals by hydrogen isotope dilution. Journal of Applied Physiology, 69, 885–891. 10.1152/jappl.1990.69.3.885 2246176

[ece36301-bib-0132] Robbins, C. (2012). Wildlife feeding and nutrition. New York ,NY: Academic Press.

[ece36301-bib-0133] Ryan, C. , McHugh, B. , O’Connor, I. , & Berrow, S. (2013). Lipid content of blubber biopsies is not representative of blubber in situ for fin whales (Balaenoptera physalus). Marine Mammal Science, 29, 542–547.

[ece36301-bib-0134] Ryg, M. , Smith, T. G. , & Øritsland, N. A. (1988). Thermal Significance of the Topographical Distribution of Blubber in Ringed Seals (Phoca hispida). Canadian Journal of Fisheries and Aquatic Sciences, 45, 985–992.

[ece36301-bib-0135] Ryser‐Degiorgis, M.‐P. (2013). Wildlife health investigations: Needs, challenges and recommendations. BMC Veterinary Research, 9, 223 10.1186/1746-6148-9-223 24188616 PMC4228302

[ece36301-bib-0136] Samuel, A. M. , & Worthy, G. A. J. (2004). Variability in fatty acid composition of bottlenose dolphin (Tursiops truncatus) blubber as a function of body site, season, and reproductive state. Canadian Journal of Zoology, 82, 1933–1942.

[ece36301-bib-0137] Sawicka‐Kapusta, K. (1974). Changes in the gross body composition and energy value of the bank voles during their postnatal development. Acta Theriologica, 19, 27–54. 10.4098/AT.arch.74-3

[ece36301-bib-0138] Schamber, J. L. , Esler, D. , & Flint, P. L. (2009). Evaluating the validity of using unverified indices of body condition. Journal of Avian Biology, 40, 49–56. 10.1111/j.1600-048X.2008.04462.x

[ece36301-bib-0139] Schmidt‐Nielsen, K. , Bolis, L. , & Taylor, C. R. (1980). Comparative physiology: Primitive mammals. Cambridge: Cambridge University Press.

[ece36301-bib-0140] Schulte‐Hostedde, A. , Millar, J. , & Hickling, G. (2001). Evaluating body condition in small mammals. Canadian Journal of Zoology, 79, 1021–1029. 10.1139/z01-073

[ece36301-bib-0141] Schulte‐Hostedde, A. I. , Zinner, B. , Millar, J. S. , & Hickling, G. J. (2005). Restitution of mass‐size residuals: Validating body condition indices. Ecology, 86, 155–163. 10.1890/04-0232

[ece36301-bib-0142] Schwarz, L. K. , Villegas‐Amtmann, S. , Beltran, R. S. , Costa, D. P. , Goetsch, C. , Hückstädt, L. , … Peterson, S. H. (2015). Comparisons and Uncertainty in Fat and Adipose Tissue Estimation Techniques: The Northern Elephant Seal as a Case Study. PLoS ONE, 10, e0131877 10.1371/journal.pone.0131877 26121044 PMC4486730

[ece36301-bib-0143] Seyboth, E. , Groch, K. R. , Dalla, R. L. , Reid, K. , Flores, P. A. C. , & Secchi, E. R. (2016). Southern right whale (Eubalaena australis) reproductive success is influenced by Krill (Euphausia superba) density and climate. Scientific Reports, 6:28205 10.1038/srep28205 27306583 PMC4910057

[ece36301-bib-0144] Shier, P. D. , & Schemmel, R. (1975). Effects of diet, age, strain and anatomical site on fat depot triglyceride and fatty acid content in rats. Proceedings of the Society for Experimental Biology and Medicine, 149, 864–870. 10.3181/00379727-149-38915 1166082

[ece36301-bib-0145] Smedes, F. (1999). Determination of total lipid using non‐chlorinated solvents. Analyst, 124, 1711–1718. 10.1039/a905904k

[ece36301-bib-0146] Smith, D. , Engel, B. , Diskin, A. M. , Španěl, P. , & Davies, S. J. (2002). Comparative measurements of total body water in healthy volunteers by online breath deuterium measurement and other near‐subject methods. The American Journal of Clinical Nutrition, 76, 1295–1301. 10.1093/ajcn/76.6.1295 12450896 PMC5207311

[ece36301-bib-0147] Speakman, J. R. (2001). Body composition analysis of animals: A handbook of non‐destructive methods. Cambridge: Cambridge University Press.

[ece36301-bib-0148] Stirling, I. , Thiemann, G. W. , & Richardson, E. (2008). Quantitative support for a subjective fatness index for immobilized polar bears. The Journal of Wildlife Management, 72, 568–574. 10.2193/2007-123

[ece36301-bib-0149] Stower, W. , Davies, D. , & Jones, I. (1960). Morphometric studies of the desert locust, Schistocerca gregaria (Forsk.). The Journal of. Animal Ecology, 29, 309–339. 10.2307/2206

[ece36301-bib-0150] Strandberg, U. , Käkelä, A. , Lydersen, C. , Kovacs, K. M. , Grahl‐Nielsen, O. , Hyvärinen, H. , & Käkelä, R. (2008). Stratification, composition, and function of marine mammal blubber: the ecology of fatty acids in marine mammals. Physiological and Biochemical Zoology, 81, 473–485. 10.1086/589108 18532882

[ece36301-bib-0151] Szabo, D. T. (2014). Transcriptomic biomarkers in safety and risk assessment of chemicals In Gupta R. C. (Ed.), Biomarkers in toxicology, Academic Press. e‐book.

[ece36301-bib-0152] Tartu, S. , Bourgeon, S. , Aars, J. , Andersen, M. , Polder, A. , Thiemann, G. W. , … Routti, H. (2017). Sea ice‐associated decline in body condition leads to increased concentrations of lipophilic pollutants in polar bears (Ursus maritimus) from Svalbard, Norway. Science of the Total Environment, 576, 409–419. 10.1016/j.scitotenv.2016.10.132 27794227

[ece36301-bib-0153] Telfer, N. , Cornell, L. , & Prescott, J. (1970). Do dolphins drink water? Journal of the American Veterinary Medical Association, 157(5):555‐558 5452073

[ece36301-bib-0154] Toedt, M. E. (2001). A histological and ultrastructural examination of the integument of Delphinus delphis. Masters Thesis: University of North Carolina at Wilmington.

[ece36301-bib-0155] Toïgo, C. , Gaillard, J.‐M. , Van Laere, G. , Hewison, M. , & Morellet, N. (2006). How does environmental variation influence body mass, body size, and body condition? Roe deer as a case study. Ecography, 29, 301–308. 10.1111/j.2006.0906-7590.04394.x

[ece36301-bib-0156] Varanasi, U. , Stein, J. E. , Tilbury, K. L. , Meador, J. P. , Sloan, C. A. , Clark, R. C. , & Chan, S.‐L. (1994). Chemical contaminants in gray whales (Eschrichtius robustus) stranded along the west coast of North America. Science of the Total Environment, 145, 29–53. 10.1016/0048-9697(94)90296-8 8016628

[ece36301-bib-0157] Víkingsson, G. A. (1995). Body condition of fin whales during summer off Iceland. Developments in Marine Biology, 4, 361–369.

[ece36301-bib-0158] Ward, E. J. , Holmes, E. E. , & Balcomb, K. C. (2009). Quantifying the effects of prey abundance on killer whale reproduction. Journal of Applied Ecology, 46, 632–640. 10.1111/j.1365-2664.2009.01647.x

[ece36301-bib-0159] Waugh, C. A. , & Monamy, V. (2016). Opposing lethal wildlife research when nonlethal methods exist: scientific whaling as a case study. Journal of Fish and Wildlife Management, 7, 231–236. 10.3996/072015-JFWM-061

[ece36301-bib-0160] Waugh, C. A. , Nichols, P. D. , Noad, M. C. , & Bengtson Nash, S. (2012). Lipid and fatty acid profiles of migrating Southern Hemisphere humpback whales Megaptera novaeangliae. Marine Ecology Progress Series, 471, 271–281. 10.3354/meps10059

[ece36301-bib-0161] Waugh, C. A. , Nichols, P. D. , Schlabach, M. , Noad, M. , & Bengtson Nash, S. M. (2014). Vertical distribution of lipids, fatty acids and organochlorine contaminants in the blubber of southern hemisphere humpback whales (*Megaptera novaeangliae*). Marine Environmental Research, 94, 24–31. 10.1016/j.marenvres.2013.11.004 24315760

[ece36301-bib-0162] Webster, T. , Dawson, S. , & Slooten, E. (2010). A simple laser photogrammetry technique for measuring Hector's dolphins (Cephalorhynchus hectori) in the field. Marine Mammal Science, 26, 296–308.

[ece36301-bib-0163] Weller, D. W. , Cockcroft, V. G. , Würsig, B. , Lynn, S. K. , & Fertl, D. (1997). Behavioral responses of bottlenose dolphins to remote biopsy sampling and observations of surgical biopsy wound healing. Aquatic Mammals, 23(1): 49‐58.

[ece36301-bib-0164] Wells, J. , & Fewtrell, M. (2006). Measuring body composition. Archives of Disease in Childhood, 91, 612–617. 10.1136/adc.2005.085522 16790722 PMC2082845

[ece36301-bib-0165] Whitehead, H. , & Payne, R. (1981). New techniques for assessing populations of right whales without killing them. Mammals in the Seas: General Papers and Large Cetaceans, 3, 189.

[ece36301-bib-0166] Wilder, S. M. , Raubenheimer, D. , & Simpson, S. J. (2016). Moving beyond body condition indices as an estimate of fitness in ecological and evolutionary studies. Functional Ecology, 30, 108–115. 10.1111/1365-2435.12460

[ece36301-bib-0167] Williams, R. , Vikingsson, G. A. , Gislason, A. , Lockyer, C. , New, L. , Thomas, L. , & Hammond, P. S. (2013) Evidence for density‐dependent changes in body condition and pregnancy rate of North Atlantic fin whales over four decades of varying environmental conditions. ICES Journal of Marine Science: Journal du Conseil, 70(6), 1273–1280. fst059.

[ece36301-bib-0168] Worthy, G. A. J. , & Edwards, E. F. (1990). Morphometric and Biochemical Factors Affecting Heat Loss in a Small Temperate Cetacean (Phocoena phocoena) and a Small Tropical Cetacean (Stenella attenuata). Physiological Zoology, 63, 432–442. 10.1086/physzool.63.2.30158506

[ece36301-bib-0169] Yordy, J. E. , Pabst, D. A. , McLellan, W. A. , Wells, R. S. , Rowles, T. K. , & Kucklick, J. R. (2010). Tissue‐specific distribution and whole‐body burden estimates of persistent organic pollutants in the bottlenose dolphin (Tursiops truncatus). Environmental Toxicology and Chemistry, 29, 1263–1273. 10.1002/etc.152 20821568

[ece36301-bib-0170] Zeng, X. , Ji, J. , Hao, Y. , & Wang, D. (2015). Topographical distribution of blubber in finless porpoises (Neophocaena asiaeorientalis sunameri): A result from adapting to living in coastal waters. Zoological Studies, 54, 1 10.1186/s40555-015-0111-1 PMC6661438 31966119

[ece36301-bib-0171] Zhang, D. , van der Hoop, J. M. , Petrov, V. , Rocho‐Levine, J. , Moore, M. J. , & Shorter, K. A. (2019). Simulated and experimental estimates of hydrodynamic drag from bio‐logging tags. Marine Mammal Science. 10.1111/mms.12627

